# Sub-nanometer Copper Clusters as Alternative Catalysts for the Selective Oxidation of Methane to Methanol with Molecular O_2_

**DOI:** 10.1021/acs.jpca.2c02895

**Published:** 2022-07-21

**Authors:** Mario Gallego, Avelino Corma, Mercedes Boronat

**Affiliations:** Instituto de Tecnología Química (UPV-CSIC), Universitat Politècnica de València − Consejo Superior de Investigaciones Científicas, Avda. de los Naranjos s/n, 46022 Valencia, Spain

## Abstract

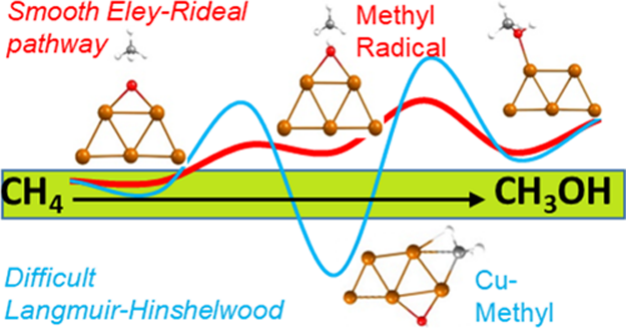

The partial oxidation of methane to methanol with molecular O_2_ at mild reaction conditions is a challenging process, which is efficiently catalyzed in nature by enzymes. As an alternative to the extensively studied Cu-exchanged zeolites, small copper clusters composed by just a few atoms appear as potential specific catalysts for this transformation. Following previous work in our group that established that the reactivity of oxygen atoms adsorbed on copper clusters is closely linked to cluster size and morphology, we explore by means of DFT calculations the ability of bidimensional (2D) and three-dimensional (3D) Cu_5_ and Cu_7_ clusters to oxidize partially methane to methanol. A highly selective Eley–Rideal pathway involving homolytic C–H bond dissociation and a non-adsorbed radical-like methyl intermediate is favored when bicoordinated oxygen atoms, preferentially stabilized at the edges of 2D clusters, are available. Cluster morphology arises as a key parameter determining the nature and reactivity of adsorbed oxygen atoms, opening the possibility to design efficient catalysts for partial methane oxidation based on copper clusters.

## 1 Introduction

**Scheme 1:**
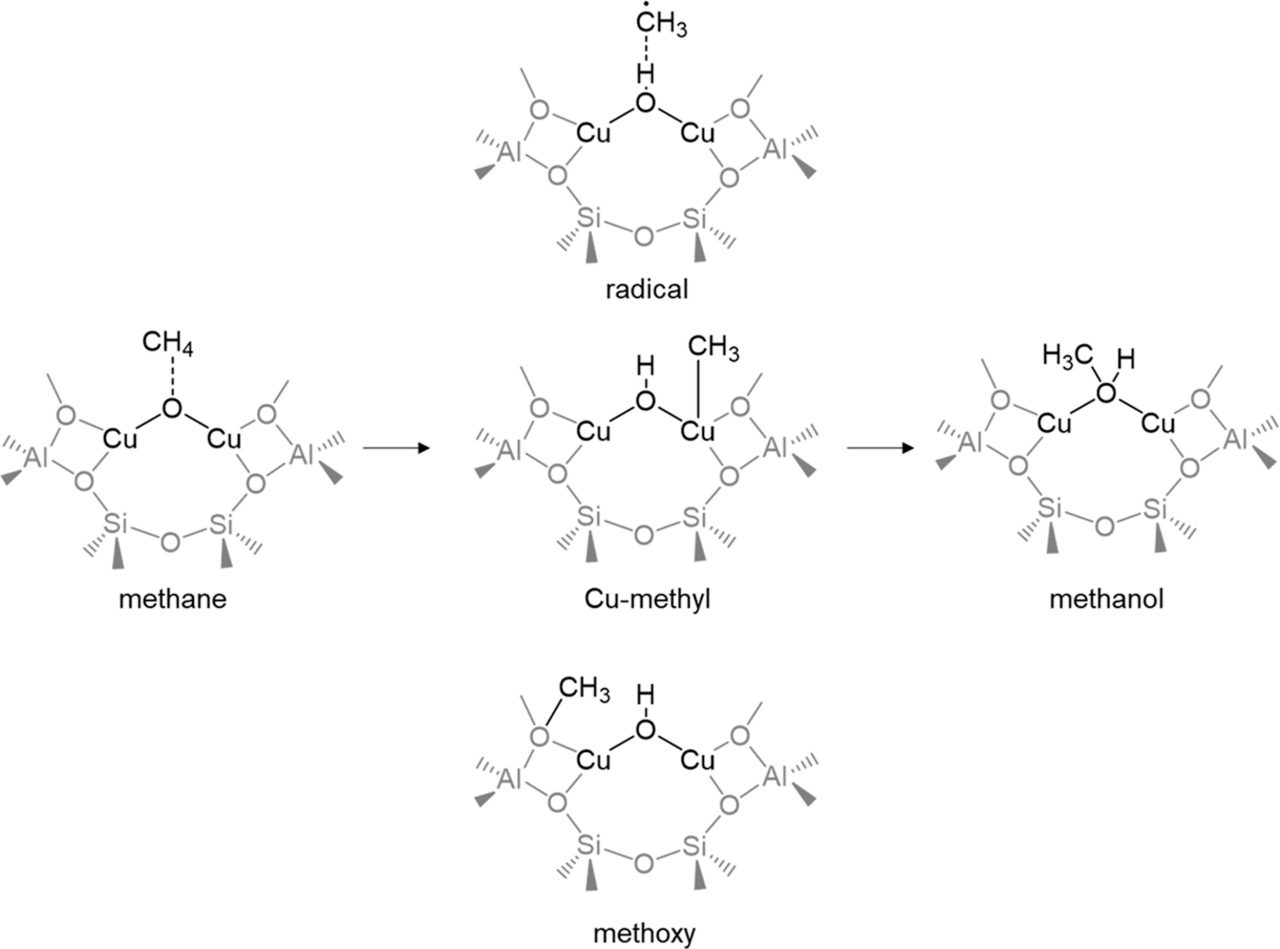
Radical and Non-radical Intermediates Proposed for Methane Oxidation to Methanol on Cu-Exchanged Zeolites

In the transition period from traditional fossil fuels to renewable energy technologies, methane has attracted great attention as raw material due to its availability, low cost, and small environmental footprint. An important incentive relies on the direct and efficient transformation of methane into liquid products, which are easier to transport, and able to act as versatile chemical feedstock. The current industrial process to upgrade methane is an indirect route based on the intermediate production of synthesis gas (CO + H_2_), which is further transformed into methanol or hydrocarbons. An appealing alternative is the partial oxidation of methane to methanol at mild reaction conditions.^1−3^ Inspired by the high selectivity afforded by the particulate methane mono-oxygenase (pMMO) enzyme, Cu-exchanged zeolites have been explored as catalysts for the partial oxidation of methane to methanol with molecular O_2_.^4−11^ This is a challenging process not only because of the high C-H bond dissociation energy of methane but especially because of the higher reactivity of the methanol product that might result in further oxidation to CO_2_. In the last decade, high selectivity to methanol has been achieved by means of either a stepwise stoichiometric process^8,9^ consisting of three consecutive steps (high-temperature (673–723 K) activation of the catalyst with O_2_, reaction of methane with the activated Cu-exchanged zeolite at 473 K to form methoxy or methanol, and final extraction of methanol with water or steam) or, more recently, through a continuous catalytic process using CH_4_, H_2_O, and O_2_ at 473 K.^10,11^ In both cases, the proposed active sites are dimeric or trimeric Cu-oxo species stabilized by the zeolite framework under the proper reaction conditions.^12−14^ Kinetic, spectroscopic, and computational studies indicate that methane C–H bond dissociation is homolytic and produces a hydroxyl group bound to Cu and a methyl intermediate that can either bind to Cu, form a framework-bound methoxy group, or yield methanol by recombination with the hydroxyl group following a radical rebound mechanism (see Scheme 1). The evolution of the methyl intermediate and, therefore, the global reactivity of the system depends on a combination of factors such as Cu speciation, Al content, zeolite topology, or reaction conditions. Intense research efforts are currently devoted to understand and control the influence of these parameters on the activity and selectivity of Cu-exchanged zeolites.^15−22^

An alternative approach to design specific catalysts for selective oxidation reactions relies on the use of small metal clusters composed of just a few atoms and exhibiting catalytic properties different from those of isolated metal cations and bulk metals.^23−32^ Previous theoretical and experimental work in our group has demonstrated that the ability of Cu clusters to dissociate O_2_ and the subsequent reactivity of the resulting adsorbed O atoms is closely linked to the atomicity and morphology of the cluster. The most stable isomers of Cu clusters composed by five atoms or less (Cu_*n*_ with *n* ≤ 5) are planar, and their electronic structure localized at the edges of the cluster leads to a high activation energy for O_2_ dissociation, which makes them more resistant against oxidation than three-dimensional isomers.^33,34^ In addition, the resulting adsorbed O atoms are usually bicoordinated and easier to transfer following Eley–Rideal mechanisms, as found theoretically for propene epoxidation^35^ and CO oxidation.^36^ Now, we extend this computational research line to the selective oxidation of methane to methanol in order to determine whether the reaction mechanism is analogous to that proposed for Cu-exchanged zeolites and to find the requirements of atomicity and morphology that could result in an improved catalytic performance. For this purpose, planar Cu_5_-2D and three-dimensional Cu_5_-3D and Cu_7_ clusters with two adsorbed O atoms have been used as catalyst models, and different pathways for the selective oxidation of methane to methanol have been explored. It has been found that bicoordinated O atoms stabilized at the edges of Cu_5_-2D clusters favor the homolytic dissociation of the C–H bond of methane and the selective formation of methanol following a radical rebound mechanism, opening the possibility to design more efficient catalysts based on supported Cu_5_ clusters.

## 2 Theoretical Methods

All calculations are based on density functional theory DFT and were performed using the VASP 5.2 code^37,38^ and the PBE functional.^39^ The valence density was expanded in a plane wave basis set with a kinetic energy cutoff of 600 eV, and the effect of the core electrons in the valence density was taken into account by means of the projected augmented wave (PAW) formalism.^40^ All calculations are spin-polarized. Electronic energies were converged to 10^–6^ eV using a Gaussian-smearing method with a width of 0.01 eV, and geometries were optimized until forces on atoms were < 0.01 eV/A. The clusters and molecules were placed in a 20 Å × 20 Å × 20 Å cubic box, large enough to avoid spurious interactions between periodically repeated systems, and integration in the reciprocal space was carried out at the Γ *k*-point of the Brillouin zone. The positions of all atoms in the system were fully optimized without any restriction, and all stationary points were characterized by frequency calculations. The Hessian matrix and vibrational frequencies were calculated using density functional perturbation theory (DFPT).^41^ Transition states were located using the DIMER^42,43^ algorithm. Atomic charges were calculated using the NBO approach.^44^ The MOLDEN^45^ and ChemCraft^46^ programs were used throughout the work to visualize the systems and their frequencies.

The absolute Gibbs free energies of all species are given by
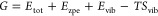
where *E*_tot_ is the electronic energy obtained from the DFT calculation, *E*_zpe_ is the zero point energy correction, *E*_vib_ is the vibrational thermal energy contribution, and *S*_vib_ is the vibrational entropy. The vibrational contributions to the energy and entropy were calculated at 478 K according to
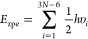

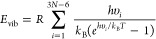

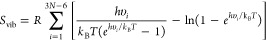
using the vibrational frequencies υ obtained from the DFT calculations.

## 3 Results and Discussion

Previous theoretical and experimental studies have demonstrated that O_2_ adsorbs on small Cu_*n*_ clusters, forming stable complexes in which the O–O bond is activated by charge transfer from the metal cluster to the π* orbital of O_2_ and facilitating its dissociation to produce adsorbed O atoms.^33,34^ In contrast, methane interaction with Cu_5_-2D, Cu_5_-3D, and Cu_7_ clusters is weak and does not lead to any activation of the C–H bonds (see Figure S1 and Table S1). Therefore, partly oxidized Cu_5_-2D, Cu_5_-3D, and Cu_7_ clusters with two adsorbed O atoms originated from O_2_ dissociation, structures **1**, **2**, and **3** in Figure S1, were taken as the starting systems to investigate the possible mechanisms of methane oxidation to methanol catalyzed by copper clusters. Both bicoordinated and three-coordinated O atoms with potentially different reactivities are present in these structures. Taking into account the possibility of weak binding of methane to the catalyst, two types of pathways were investigated for all systems: Langmuir–Hinshelwood (LH) pathways with all the reactant species adsorbed on the clusters and Eley–Rideal (ER) pathways with only one of the reactants adsorbed on the cluster and the other one reacting from the gas phase.

### 3.1 Methane Oxidation on 2D and 3D Cu_5_ Clusters

**Figure 1:**
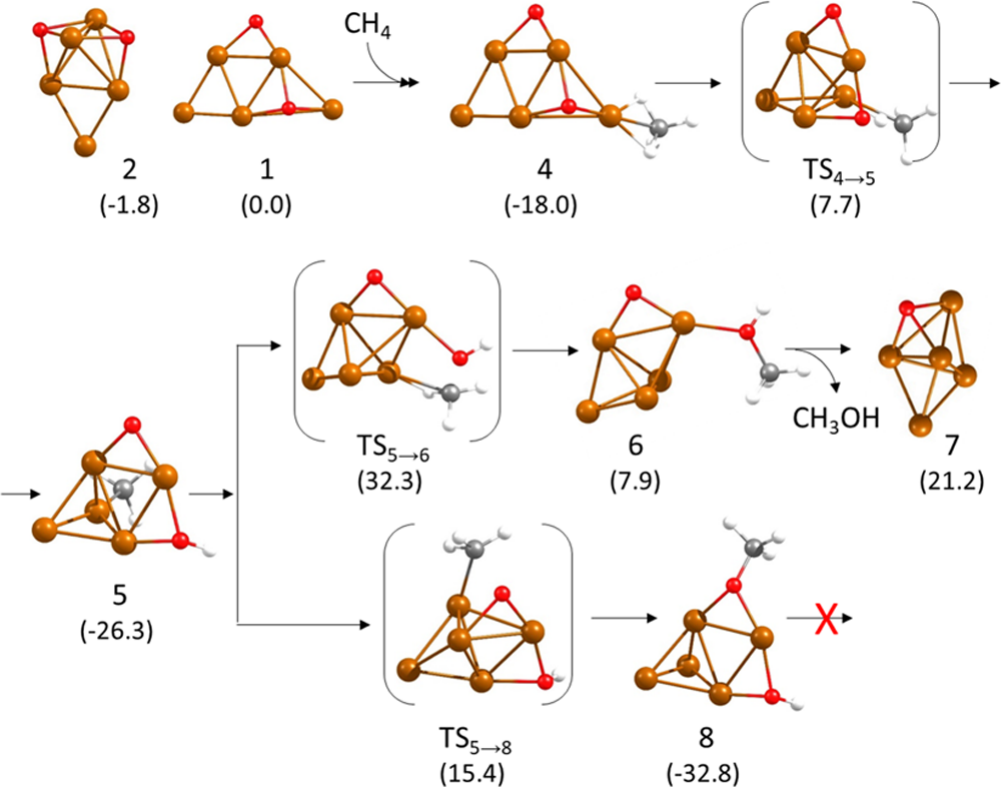
Optimized geometries of the structures involved in the first part of the mechanism of methane oxidation on Cu_5_ clusters following a Langmuir–Hinshelwood pathway. Relative energies of each structure with respect to the initial reactant **1** are given in kcal/mol in parenthesis below the structure label. Cu, O, C, and H atoms are depicted as orange, red, gray, and white balls, respectively.

In a first step, methane adsorbs on a low-coordinated Cu atom in direct contact with one of the O atoms present on the cluster (site **a** in Figure S2). Other adsorption sites with Cu only bonded to two other Cu atoms (site **b** in Figure S2) or with a higher degree of coordination (sites **c**–**e**) lead to less stable structures or to non-bonded systems. Interestingly, the interaction of methane with site **a** in the O containing 3D Cu_5_ cluster (structure **2** in Figure 1) produces its deformation into the same planar species (structure **4** in Figure 1) obtained by methane adsorption on the Cu_5_-2D cluster (structure **1**). The process is energetically favorable, and two C–H bonds of methane become slightly activated by interaction with Cu, with optimized Cu–H and C–H distances of 1.831, 1.805, 1.125, and 1.129 Å, respectively. Dissociation of one of these C–H bonds is facilitated by interaction of the H atom with the nearby O atom. The C–H and O–H distances in the transition state **TS4 → 5** are 1.397 and 1.336 Å, and the calculated activation energy is 25.7 kcal/mol. In the resulting structure **5**, the hydroxyl group is bridged between two Cu atoms at Cu–O distances of 1.887 and 1.951 Å, while the methyl group is monocoordinated on top of one Cu atom at a Cu–C distance of 1.916 Å. In order to form methanol by recombination of these two groups, the hydroxyl must break one of the Cu–O bonds in which it is involved while forming a new O–C bond with the surface methyl. The Cu–C, C–O, and O–Cu optimized distances in transition state **TS5 → 6** are 2.057, 1.837, and 1.923 Å, respectively, indicating that the C atom is still bonded to Cu while the C–O bond is being formed. This step is thermodynamically unfavorable and requires overcoming a prohibitive activation energy barrier of 58.6 kcal/mol. A competing process is the migration of the methyl group in intermediate **5** toward the adsorbed O atom to form a highly stable methoxy group (structure **8** in Figure 1). The optimized Cu–C and C–O distances in transition state **TS5 → 8** are 2.138 and 1.917 Å, respectively, and the calculated activation energy is 41.7 kcal/mol. The methoxy species **8** is 6.5 kcal/mol more stable than the methyl intermediate **5**, and it was not possible to optimize a transition state for the direct reaction between the methoxy and hydroxyl groups to form adsorbed methanol, which altogether renders the LH pathway kinetically non-accessible. This situation is analogous to that described for Cu-exchanged zeolites, where the high stability of the methoxy intermediates makes necessary an additional extraction step with water.^5,8,9^ Additional competitive processes starting from the strongly bound intermediate **5**, such as secondary C–H bond dissociation to form a CH_2_ intermediate, also involve lower activation barriers than methanol production (see Figure S3).

**Figure 2:**
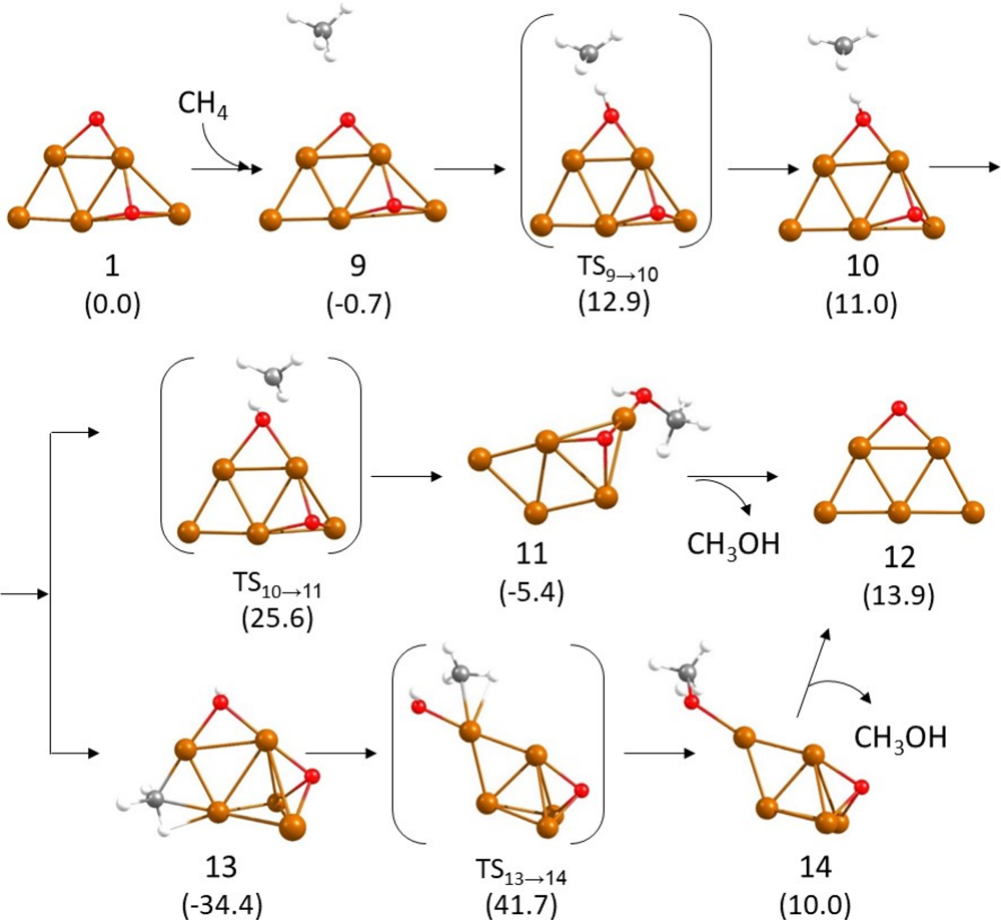
Optimized geometries of the structures involved in the first part of the mechanism of methane oxidation on Cu_5_ clusters following an Eley–Rideal pathway. Relative energies of each structure with respect to the initial reactant **1** are given in kcal/mol in parenthesis below the structure label. Cu, O, C, and H atoms are depicted as orange, red, gray, and white balls, respectively.

An alternative ER pathway was explored starting from methane physisorbed close to an O atom of structure **1**. The interaction with the bicoordinated O atom is weak, only −0.7 kcal/mol, and the shortest H–O distance in the resulting complex is 2.564 Å (see structure **9** in Figure 2). However, an H transfer from CH_4_ to the O atom is energetically accessible with a calculated activation energy of 13.6 kcal/mol, yielding a metastable structure **10** in which the resulting methyl group is not interacting with the Cu cluster. The C–H and H–O distances evolve from 1.440 and 1.128 in **TS9 → 10** to 2.104 and 0.986 in the intermediate **10**, evidencing the transfer of one of the H atoms to form a hydroxyl group. The nearly planar geometry of the non-interacting CH_3_ fragment suggests a radical nature corroborated by a simple Bader analysis of the atomic charges (see Table S2). The net atomic charges on the O and H atoms of the hydroxyl groups formed in the first step of both the LH and the ER pathways are similar, around −1*e* for O and ∼0.4*e* for H (see Table S2). In contrast, the net atomic charge on the C atom and the total charge on the methyl group are clearly negative in structures **TS4 → 5** and **5**, in which the methyl group is attached to Cu, and close to neutral in structures **TS9 → 10** and **10** with a non-interacting methyl group. Attempts to obtain a similar pathway involving the three-coordinated O atom of structure **1** failed, and H transfer always resulted in the reverse formation of a methane molecule, evidencing the importance of the coordination of O atoms on their reactivity. It should be remarked that the inclusion of dispersion forces does not modify these results due to the small size of the clusters used as catalysts (see Figure S4 in the Supporting Information.)

The radical-like intermediate **10** can evolve following three different routes. On one hand, attack of the methyl group to the bridged hydroxyl group in transition state **TS10 → 11** results in formation of adsorbed methanol (structure **11** in Figure 2) with a calculated activation energy of only 14.6 kcal/mol. Methanol desorption from structure **11** does not require additional water, as is the case for Cu-exchanged zeolites, and leaves a highly stable planar Cu_5_ cluster with an O atom adsorbed on its edge (structure **12** in Figure 2). On the other hand, the unstable methyl group of structure **10** might directly adsorb on the Cu_5_ cluster, forming either highly stable structures like **13**, with the C atom of the methyl group bridged between two Cu atoms (see Figure 2), or a similarly stable methoxy intermediate as that in structure **8** (see Figure 1). Formation of methanol from intermediate **13** requires the partial decoordination of both methyl and hydroxyl groups in transition state **TS13 → 14**, resulting in an extremely demanding activation energy barrier of 76.1 kcal/mol.

**Figure 3:**
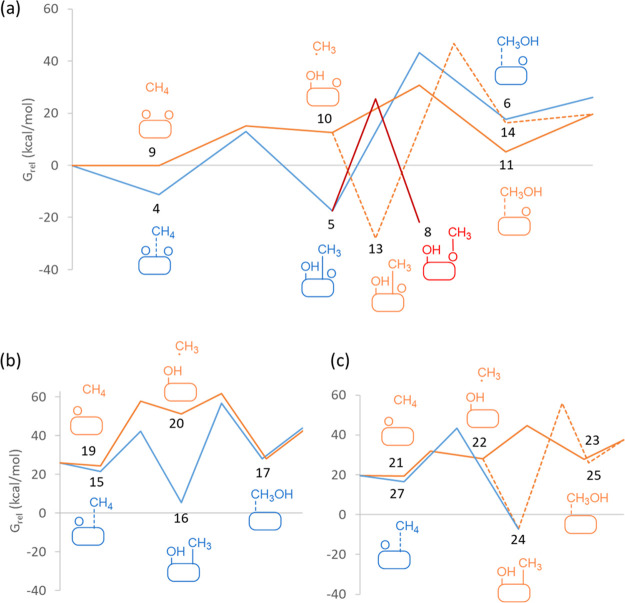
Gibbs energy profiles for CH_4_ oxidation to CH_3_OH on (a) Cu_5_-2O, (b) Cu_5_-3D-O, and (c) Cu_5_-2D-O clusters at 478 K. Langmuir–Hinshelwood and Eley–Rideal pathways are plotted as blue and orange lines, respectively. Formation of methoxy intermediates is depicted in red. The labels correspond to the optimized structures shown in Figures 1, 2, 4, and 5. The origin of Gibbs energies is in all cases the initial reactant structure **1**, and the relative Gibbs energy values are summarized in Table S3.

The results presented up to here indicate that methane activation on O containing Cu_5_ clusters via a LH mechanism is energetically accessible, but recombination of the resulting adsorbed methyl or methoxy groups with hydroxyl groups to form methanol is kinetically forbidden. Alternatively, an ER pathway in which methane reacts from gas phase through a radical-like methyl intermediate is energetically affordable, with calculated activation energies always below 15 kcal/mol. This is clearly observed in the Gibbs free energy profiles at 478 K plotted in Figure 3a, with the smoothest profile (full orange line) corresponding to the ER pathway through a radical intermediate.

**Figure 4:**
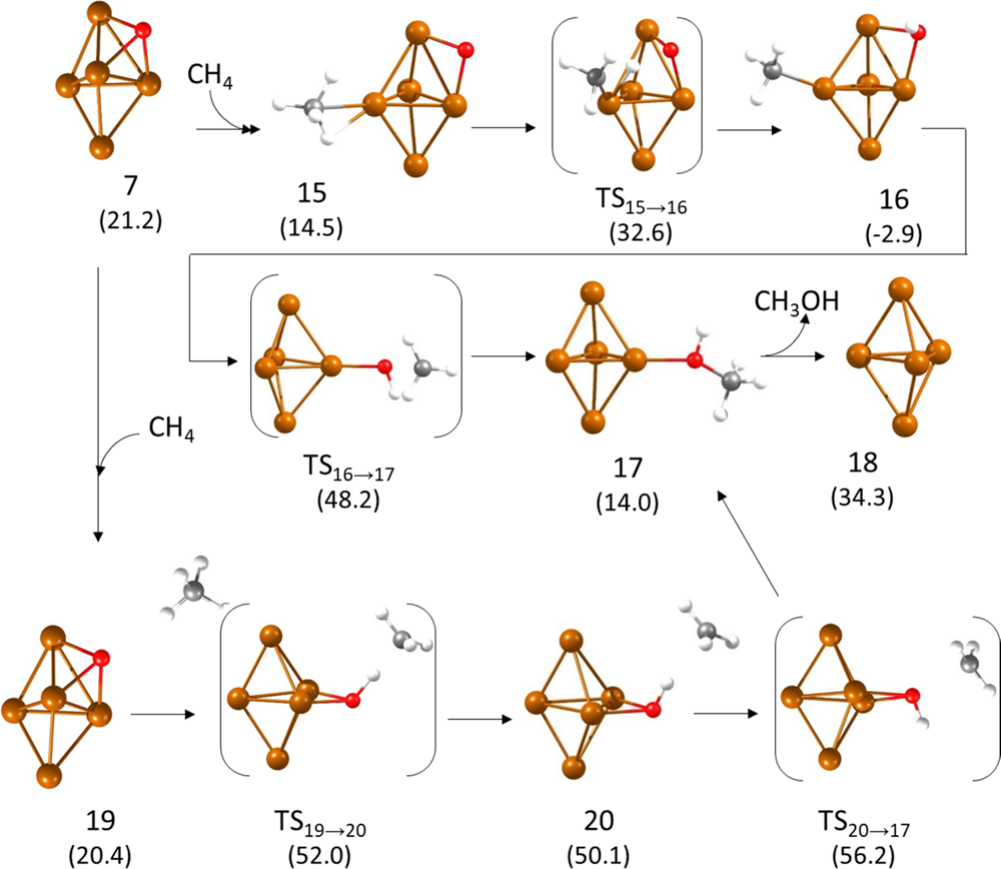
Optimized geometries of the structures involved in the second part of the mechanism of methane oxidation on a 3D Cu_5_ cluster. Relative energies of each structure with respect to the initial reactant **1** are given in kcal/mol in parenthesis below the structure label. Cu, O, C, and H atoms are depicted as orange, red, gray, and white balls, respectively.

**Figure 5:**
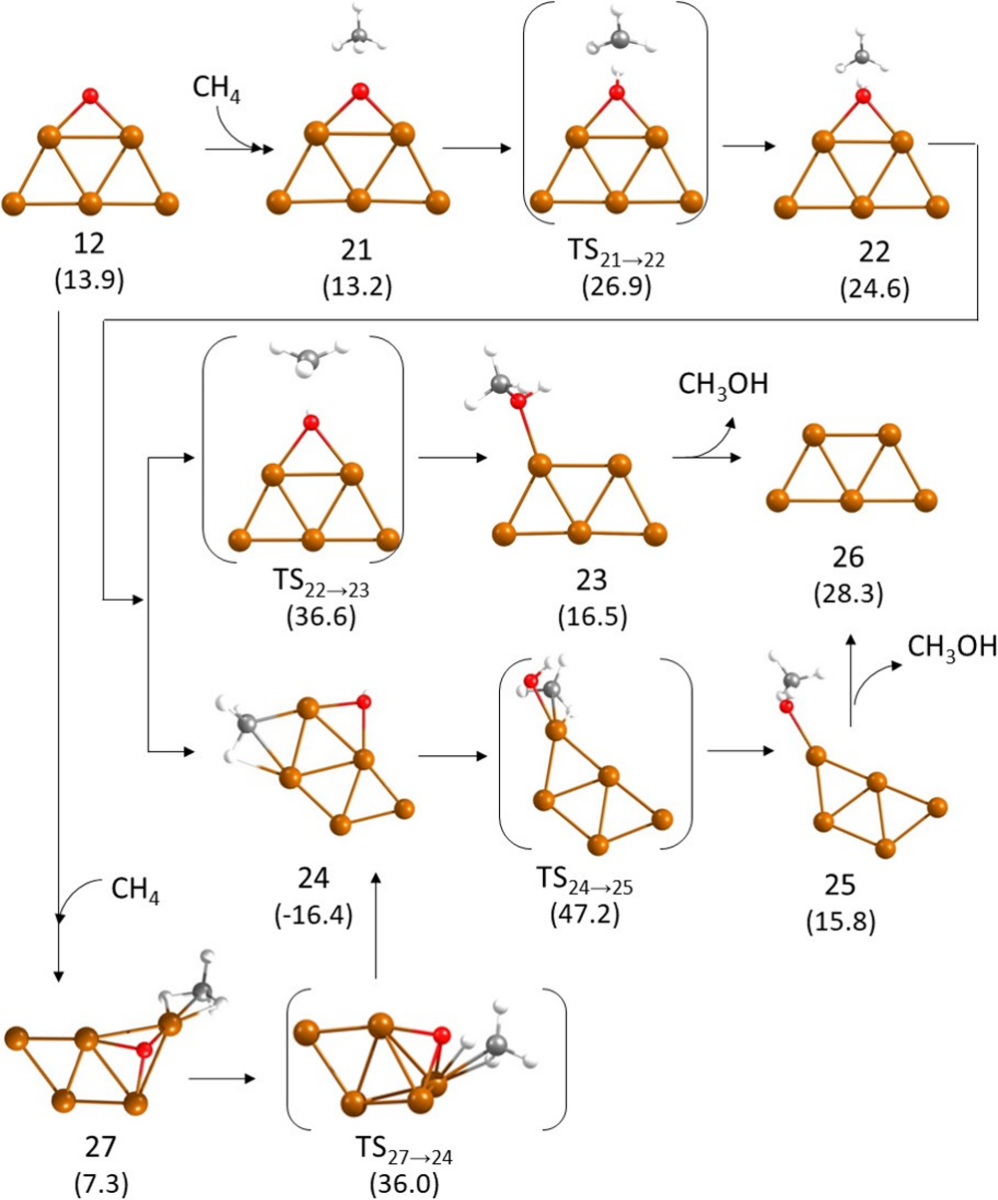
Optimized geometries of the structures involved in the second part of the mechanism of methane oxidation on a planar Cu_5_ cluster. Only the ER pathway is shown. Relative energies of each structure with respect to the initial reactant **1** are given in kcal/mol in parenthesis below the structure label. Cu, O, C, and H atoms are depicted as orange, red, gray, and white balls, respectively.

After the first cycle converting methane into methanol, only one O atom remains adsorbed on either a 3D (structure **7**) or a 2D (structure **12**) Cu_5_ cluster, ready for a second catalytic cycle. The calculated Gibbs free energy profiles for the second part of the catalytic cycle on Cu5-3D-O and Cu5-2D-O systems are plotted in Figure 3b,c, respectively, and the optimized geometries of the structures involved are shown in Figures 4 and 5. Methane adsorbs preferentially on an equatorial Cu atom of the 3D-Cu_5_ cluster, not in direct contact with O (structure **15** in Figure 4). The H transfer from methane to O through transition state **TS15 → 16** involves the bending of both adsorbed groups but not the rupture of any Cu–O or Cu–C interaction. As a consequence, the calculated activation energy is moderate (18.1 kcal/mol). The optimized C–H and H–O distances are 1.379 and 1.420 Å, respectively, similar to those obtained for **TS4 → 5**. The methyl group in intermediate **16** is monocoordinated to a Cu atom, and the hydroxyl group is bicoordinated on a cluster edge. The compact geometry of the Cu_5_ cluster forces the rupture of one Cu–O bond and the detachment of the methyl group to allow the formation of methanol through transition state **TS16 → 17**. This is the reason for the high activation energy obtained for this step, 51.1 kcal/mol, which makes again the LH pathway kinetically difficult. The alternative ER mechanism starts with a less stable reactant structure **19** in which methane does not interact with the Cu_5_ cluster. The H transfer to the only O atom in the system through **TS19 → 20** requires a high activation energy of 31.6 kcal/mol and produces a metastable methyl radical intermediate **20** (see charge distribution in Table S2) placed at a C–H distance of 2.152 Å. This methyl radical can directly react with the bridged hydroxyl group through **TS20 → 17** to form adsorbed methanol **17** with a low activation barrier of 6.1 kcal/mol or adsorb on the cluster, yielding the previously described stable structure **16**. In such case, the LH pathway would be followed with a high activation barrier for the methanol formation step.

Finally, we explored the reactivity of the 2D Cu_5_ cluster with one O atom bridged on the short edge (structure **12** in Figure 5). Taking into account that CH_4_ does not adsorb on the Cu atoms in direct contact with a bridged O atom (see Figure S2) and that CH_4_ adsorbed on the bicoordinated Cu atoms on the long edge of Cu_5_-2D is too far from the O atom to react, only the ER pathway is in principle geometrically accessible on this system.

Methane interaction with the O atom in structure **21** favors the H transfer step that initiates the ER mechanism, yielding intermediate **22** with a bicoordinated hydroxyl group and a methyl radical (see atomic charges in Table S2). The activation energy for this H transfer is as low as 13.7 kcal/mol, and the process is endothermic by 11.4 kcal/mol. The C–H-optimized distances in **TS21 → 22** and intermediate **22** are 1.442 and 2.108 Å, respectively, similar to those found for the equivalent structures **TS9 → 10** (1.440 Å) and **10** (2.104 Å) in the first part of the cycle and shorter than in **TS19 → 20** (1.496 Å) and **20** (2.152 Å) on the 3D-Cu_5_ cluster. Again, direct methanol formation through transition state **TS22 → 23** requires a low activation energy of 12 kcal/mol but competes with the adsorption of the methyl group on a Cu atom leading to structure **24**. The high stability of this intermediate containing both methyl and hydroxyl groups bicoordinated at the cluster edges implies an activation energy barrier of 63.6 kcal/mol to recombine the fragments and form adsorbed methanol (structure **25** in Figure 5), thus preventing the contribution of a LH pathway to methane conversion.

Interestingly, by forcing the adsorption of methane on a Cu atom of the Cu_5_-2D-O system, structure **27** with the O atom three-coordinated on a facet of the cluster and close to adsorbed CH_4_ was obtained. This structure is only 6 kcal/mol more stable than the weakly interacting intermediate **21** involved in the ER pathway, and the activation energy required for C–H bond dissociation through **TS27 → 24** yielding adsorbed methyl and hydroxyl moieties (structure **24** previously described) is clearly higher (28.8 kcal/mol versus 13.7 kcal/mol for C–H bond rupture through **TS21 → 22**). Therefore, it can be concluded that 2D Cu_5_ clusters and, particularly, the bicoordinated O atoms adsorbed at their edges favor the ER pathway via radical intermediates.

To facilitate comparison of all the pathways described so far, the Gibbs free energy profiles at 478 K for all the processes involving atomic O are plotted together in Figure 3, and the activation energies and Gibbs free energies obtained for the dissociation of the C–H bond in CH_4_ and for the formation of CH_3_OH by C–O bond formation are summarized in Table 1.

**Table 1:** Comparison of Langmuir–Hinshelwood (LH) and Eley–Rideal (ER) Pathways on Different Cu_5_ and Cu_7_ Clusters^a^

cluster	pathway	*E*_act_(CH) (kcal/mol)	*E*_act_(CO) (kcal/mol)	*G*_act_(CH) (kcal/mol)	*G*_act_(CO) (kcal/mol)
Cu_5_-2O	LH	25.7	58.6	24.2	60.7
Cu_5_-3D-O	LH	18.1	51.1	20.7	51.2
Cu_5_-2D-O	LH	28.8	63.6	26.8	63.3
Cu_5_-2D-O_2_	LH				
Cu_7_-2O	LH	20.6	55.9	19.8	50.8
Cu_7_-O	LH	25.7	52.7	23.9	50.9
Cu_5_-2O	ER	13.7	14.7	15.1	18.1
Cu_5_-3D-O	ER	31.6	6.2	33.7	10.6
Cu_5_-2D-O	ER	13.7	12.0	12.5	16.7
Cu_5_-2D-O_2_	ER	36.9	3.0	37.5	3.9
Cu_7_-2O	ER	14.3	9.9	13.4	14.2
Cu_7_-O	ER	30.1	5.6	27.4	7.8
Cu_7_-4O	ER	13.5	7.5	14.1	11.8

a Activation energies (*E*_act_) and Gibbs free energies (*G*_act_) at 478 K for the C–H dissociation in CH_4_ (CH) and for CH_3_OH formation (CO) steps are given in kcal/mol.

For Cu_5_ clusters with two adsorbed O atoms (Figure 3a) and for Cu_5_-3D with one adsorbed O atom (Figure 3b), LH pathways in which methane interacts with Cu before reacting (blue lines in Figure 3b) involve high Gibbs activation energies for the dissociation of the first C–H bond (∼20 kcal/mol) and unaffordable Gibbs activation energies (>50 kcal/mol) for the formation of methanol. The high stability of the methyl and hydroxyl fragments generated in the first step of the mechanism, which are in many cases bicoordinated to two Cu atoms of the cluster, explains the large energies necessary to detach these two CH_3_ and OH groups from Cu and combine them to form methanol as well as the difficult formation of methoxy groups by reaction of methyl with adsorbed O. In contrast, the alternative ER pathway according to which methane reacts from the gas phase, forming a hydroxyl group attached to Cu and a radical-like methyl intermediate (orange lines in Figure 3), requires lower Gibbs activation energies both for C–H bond dissociation (∼15 kcal/mol) and C–O bond formation (∼18 kcal/mol). However, this is only valid when the O atom participating in the reaction is bicoordinated to the Cu_5_ cluster. For the Cu_5_-3D-O system, with the O atom initially three-coordinated on a face of the cluster, the calculated activation energy for C–H bond scission is high (34 kcal/mol). The reason is that, in order to react with CH_4_, the adsorbed O atom must migrate from the face to the edge of the cluster, thus decreasing its coordination to Cu atoms from 3 to 2.

**Figure 6:**
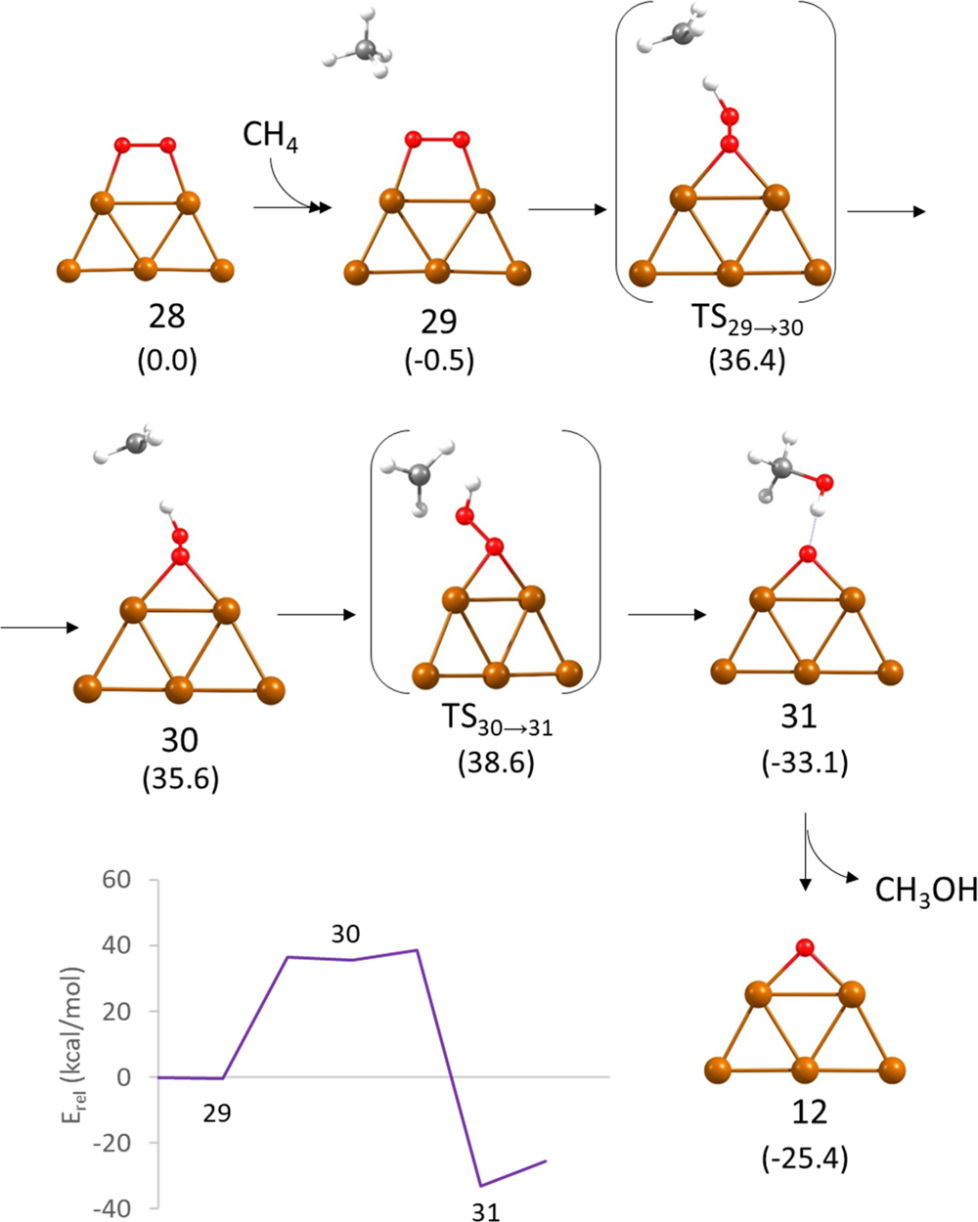
Optimized geometries of the structures involved in the mechanism of methane oxidation with molecular O_2_ on a planar Cu_5_ cluster following a ER pathway and Gibbs free energy profile at 478 K. Relative energies of each structure with respect to the initial reactant **28** are given in kcal/mol in parenthesis below the structure label. Cu, O, C, and H atoms are depicted as orange, red, gray, and white balls, respectively.

At this point, and taking into account that O_2_ dissociation on 2D Cu_5_ clusters involves a higher activation energy than on 3D Cu_5_ clusters,^33,34^ the possibility of CH_4_ oxidation by adsorbed molecular O_2_ was also investigated. Starting from O_2_ adsorbed in a bridge mode on Cu_5_ 2D (structure **28** in Figure 6), the dissociation of a C–H bond of weakly interacting methane (structure **29**) through transition state **TS29 → 30** requires surpassing an activation energy barrier of 36.9 kcal/mol and yields a radical methyl group (see charge distribution in Table S2) and a hydroperoxide group bonded to Cu_5_. From this metastable intermediate **30**, subsequent formation of methanol through transition state **TS30 → 31** is kinetically easy and thermodynamically favored, with the adsorbed methanol product **31** being 68.7 kcal/mol more stable than intermediate **30**. The high activation energy required in the first step (see Table 1) makes this route via molecular O_2_ unlikely.

### 3.2 Methane Oxidation on Cu_7_ Clusters

**Figure 7:**
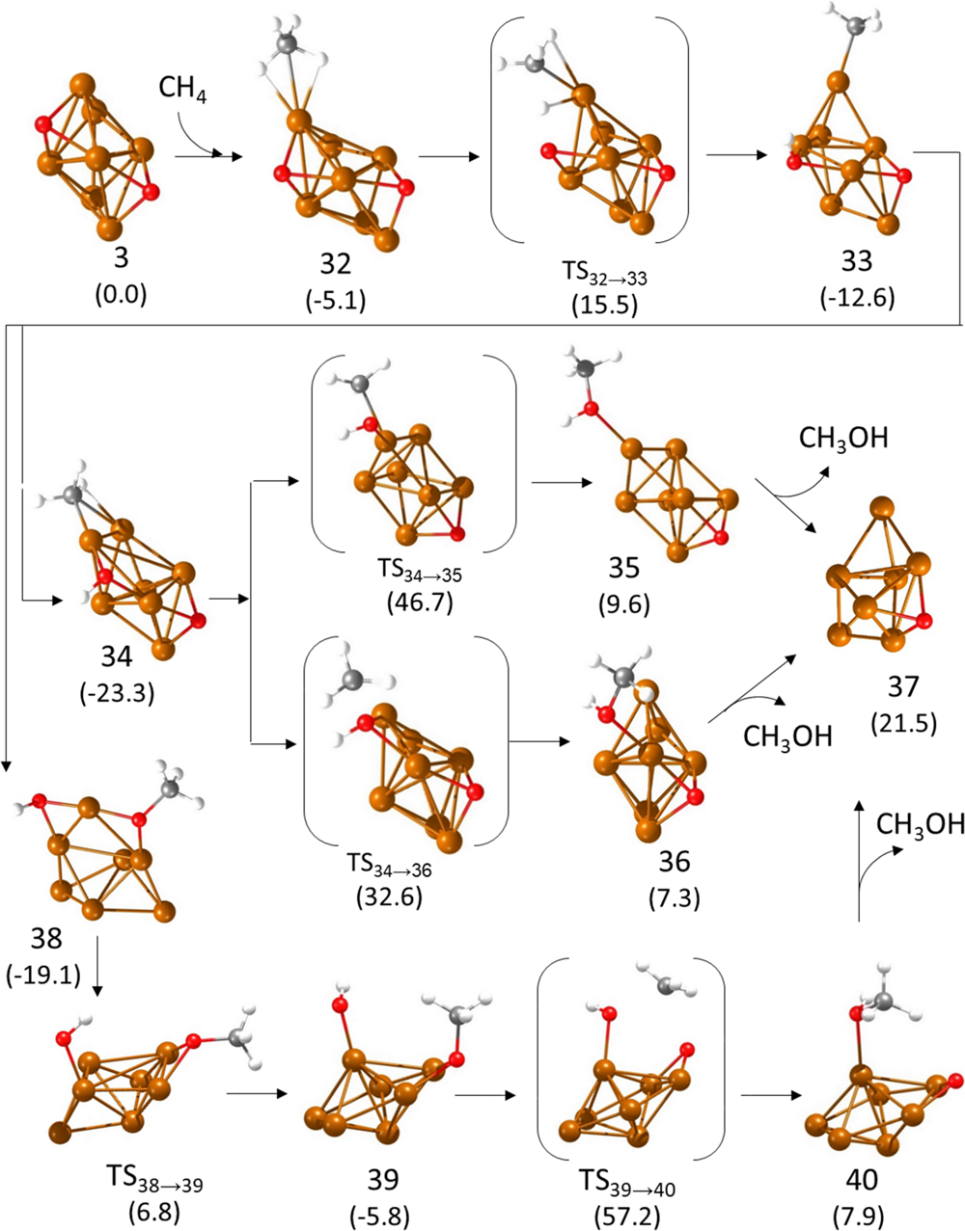
Optimized geometries of the structures involved in the first part of the mechanism of methane oxidation on Cu_7_ clusters following a Langmuir–Hinshelwood pathway. Relative energies of each structure with respect to the initial reactant **3** are given in kcal/mol in parenthesis below the structure label. Cu, O, C, and H atoms are depicted as orange, red, gray, and white balls.

Once the key aspects of the mechanism are established for 2D and 3D Cu_5_ systems, the influence of cluster size was analyzed by considering a Cu_7_ cluster with 3D morphology and with two O atoms adsorbed on opposite facets in a three-fold coordination (structure **3** in Figures S1, S7, and S8). As described for Cu_5_-3D, methane adsorbs on a low-coordinated Cu atom in direct contact with one of the O atoms present on the cluster, forming structure **32** in Figure 7, with optimized Cu–H and C–H distances of 1.979, 1.902, 1.113, and 1.120 Å, respectively. The presence of the nearby O atom facilitates the dissociation of one of the C–H bonds via transition state **TS32 → 33** with a calculated activation energy of 20.6 kcal/mol, slightly lower than that obtained for Cu_5_-3D. The C–H and O–H distances in transition state **TS32 → 33** are 1.395 and 1.322 Å, respectively, and the resulting intermediate **33** contains a methyl group monocoordinated on top of a Cu atom with a Cu–C distance of 1.917 Å and a hydroxyl group bridged between two Cu atoms. This structure evolves rapidly either to a 10.7 kcal/mol more stable intermediate **34**, in which the methyl group is also bicoordinated to two Cu atoms, or to a 6.5 kcal/mol more stable intermediate **38** with formation of a methoxy group.

From **34**, the recombination of hydroxyl and methyl groups to form methanol requires either the rupture of one Cu–O bond and one Cu–C in **TS34 → 35** or the displacement of the methyl group toward the adsorbed hydroxyl in **TS34 → 36**. In both cases, the calculated activation barriers are extremely high (70.0 kcal/mol to form **35** and 55.9 kcal/mol to form **36**), suggesting a bad performance of Cu_7_ in the oxidation of methane.

From **38**, rupture of one of the two Cu–O bonds stabilizing the hydroxyl group involves a lower activation energy of 25.9 kcal/mol, and the resulting monocoordinated hydroxyl group in intermediate **39** enables the migration of the methyl fragment to form methanol (structure **40** in Figure 7). However, the calculated activation energy for this methyl shift via transition state **TS39 → 40** in which the methyl group is at 2.180 and 2.235 Å from the two O atoms is really high (63.0 kcal/mol), again preventing the formation of methanol.

**Figure 8:**
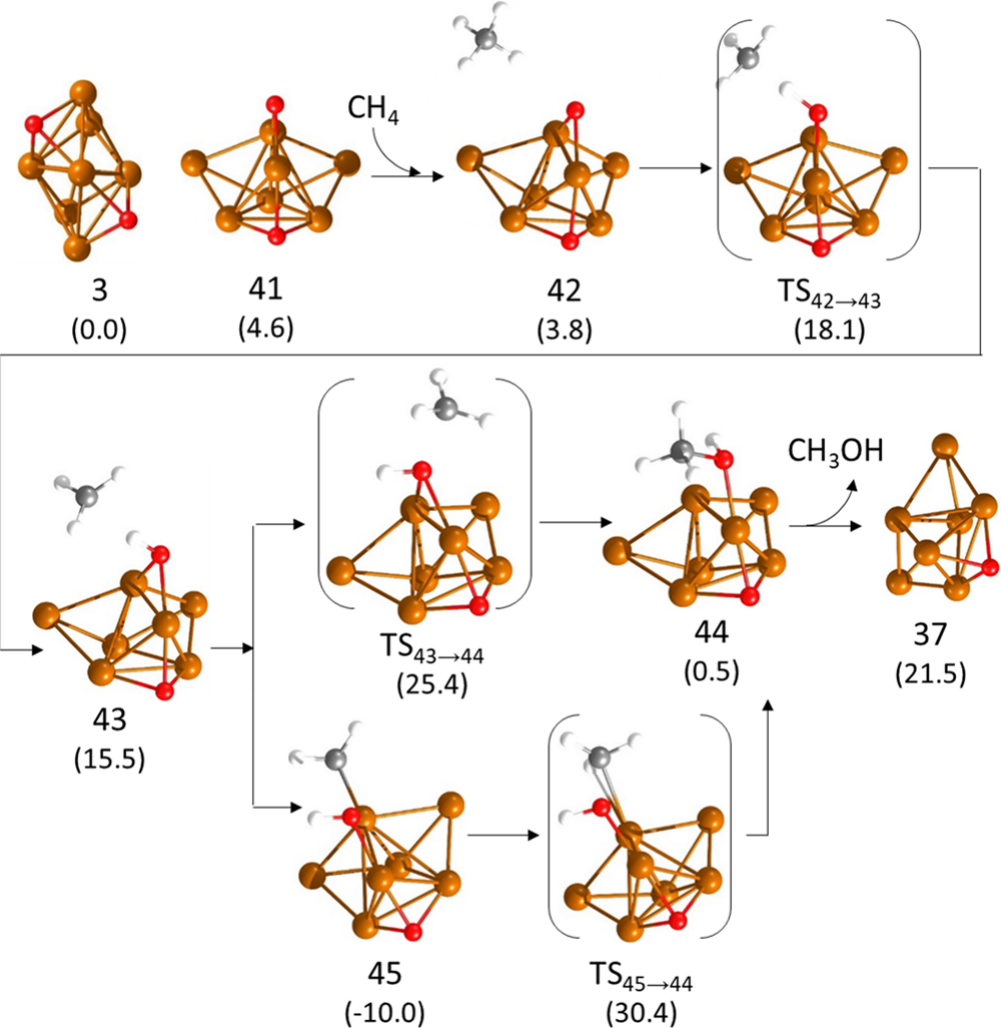
Optimized geometries of the structures involved in the first part of the mechanism of methane oxidation on Cu_7_ clusters following a Eley–Rideal pathway. Relative energies of each structure with respect to the initial reactant **3** are given in kcal/mol in parenthesis below the structure label. Cu, O, C, and H atoms are depicted as orange, red, gray, and white balls, respectively.

A closer inspection to the geometry and charge distribution of **TS34 → 36** indicates that it might be involved in an ER pathway, but it was not possible to stabilize a radical methyl intermediate connected to this transition state. It was possible, however, to obtain a complete ER mechanism starting from a Cu_7_ cluster with two adsorbed O atoms, one of them bicoordinated to only two Cu atoms (structure **41** in Figure 8). This displacement of an adsorbed O atom from a facet to an edge of the cluster is endothermic by 4.6 kcal/mol but facilitates an alternative pathway for methane oxidation on Cu_7_. As described before for Cu_5_, starting from methane physisorbed close to the bicoordinated O atom (structure **42** in Figure 8), an H transfer from CH_4_ to O yields a metastable methyl radical not in direct contact with any Cu atom. The C–H and H–O distances in **TS42 → 43** (1.436 and 1.128 Å, respectively) are similar to those in **TS8 → 9**, and the calculated activation and reaction energies for this step in Cu_7_ are 14.3 and 11.7 kcal/mol, respectively. Direct reaction of the methyl radical with the adsorbed hydroxyl produces adsorbed methanol (structure **44**) through a transition state **TS43 → 44** with a low activation energy of only 9.9 kcal/mol. However, the methyl radical fragment in intermediate **43** could also bind to one Cu atom of the cluster, forming a 25.5 kcal/mol more stable structure **45**. As in previous examples, the recombination of one methyl and one hydroxyl group, both of them anchored to Cu, involves necessarily the rupture of some Cu–O or Cu–C bonds with the corresponding energy cost. Therefore, the calculated activation energies for this type of step are high, and for the particular transformation of **45** into adsorbed methanol (structure **44**), we obtain an activation barrier of 40.4 kcal/mol.

**Figure 9:**
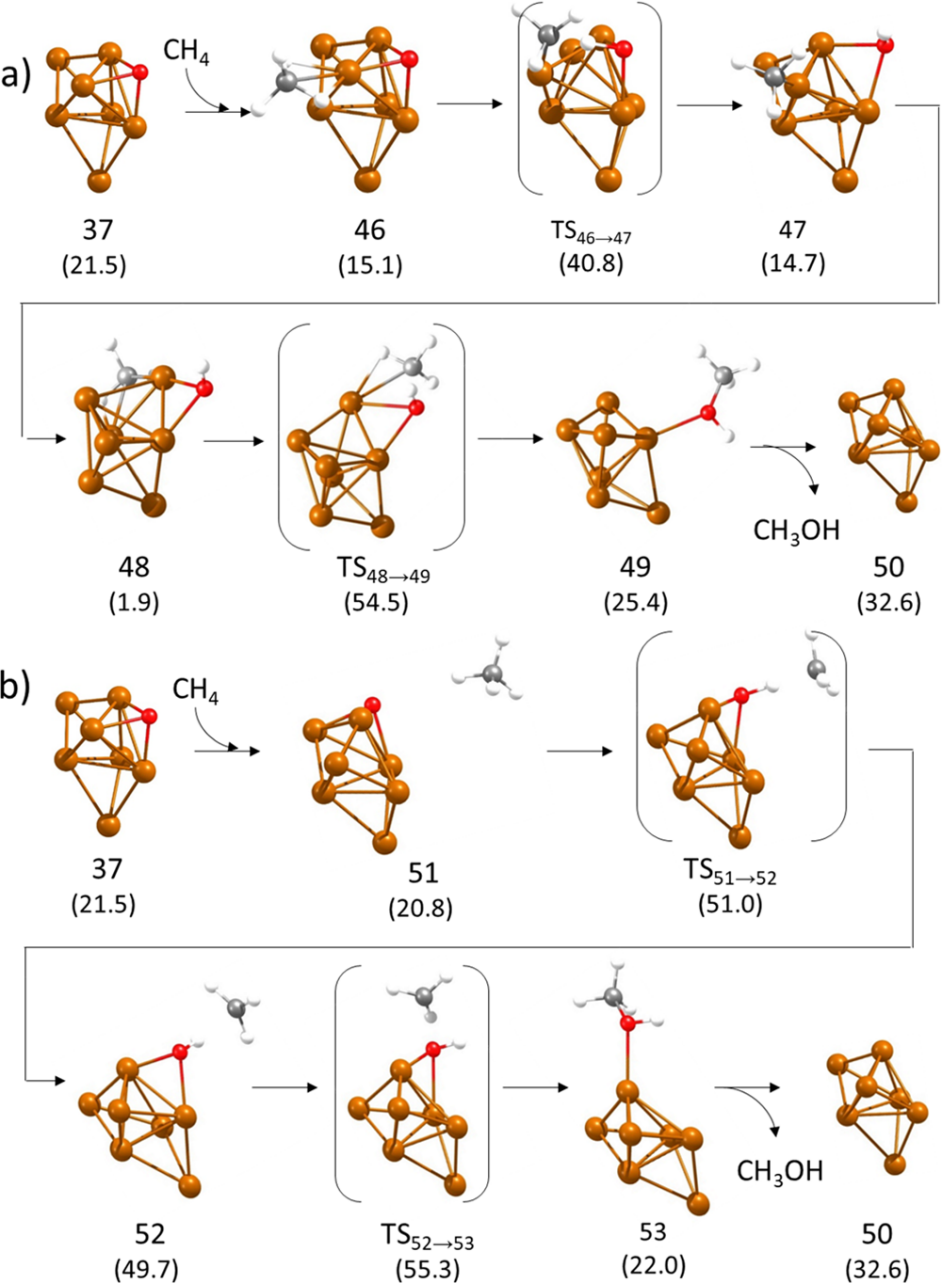
Optimized geometries of the structures involved in the second part of the mechanism of methane oxidation on Cu_7_ clusters following (a) Langmuir–Hinshelwood and (b) Eley–Rideal pathways. Relative energies of each structure with respect to the initial reactant **3** are given in kcal/mol in parenthesis below the structure label. Cu, O, C, and H atoms are depicted as orange, red, gray, and white balls, respectively.

After the first catalytic cycle, structure **37** with one O atom adsorbed on a facet is obtained irrespective of the pathway followed. To complete the computational study, LH and ER mechanisms were explored for methane oxidation on structure **37**. The LH pathway is similar to that previously described for Cu_7_ with two adsorbed O atoms. CH_4_ adsorbs on a Cu atom in direct contact with the adsorbed O (structure **46** in Figure 9a), which facilitates the C–H bond dissociation through **TS46 → 47**. A system with a monocoordinated methyl and a bicoordinated hydroxyl group (structure **47**) is initially formed, which evolves to a more stable complex **48** in which the two reactant groups occupy bridge positions between two Cu atoms. The activation energy for the C–H bond breaking step is relatively high (25.7 kcal/mol), but the subsequent recombination of fragments to form methanol via **TS48 → 49** is energetically forbidden, with a calculated barrier of 52.6 kcal/mol. On the other hand, the alternative ER pathway according to which methane reacts from the gas phase (structure **51** in Figure 9b) transferring a H to adsorbed O and producing a non-adsorbed methyl radical (structure **52** in Figure 9b) also involves a high activation energy of 30.2 kcal/mol. The reason is that the adsorbed O atom is three-coordinated, and one of the Cu–O bonds must be broken to accept the H atom and form the adsorbed hydroxyl. The subsequent reaction of the methyl radical with the bridged hydroxyl is easier and only 5.6 kcal/mol is required to form adsorbed methanol through transition state **TS52 → 53.**

**Figure 10:**
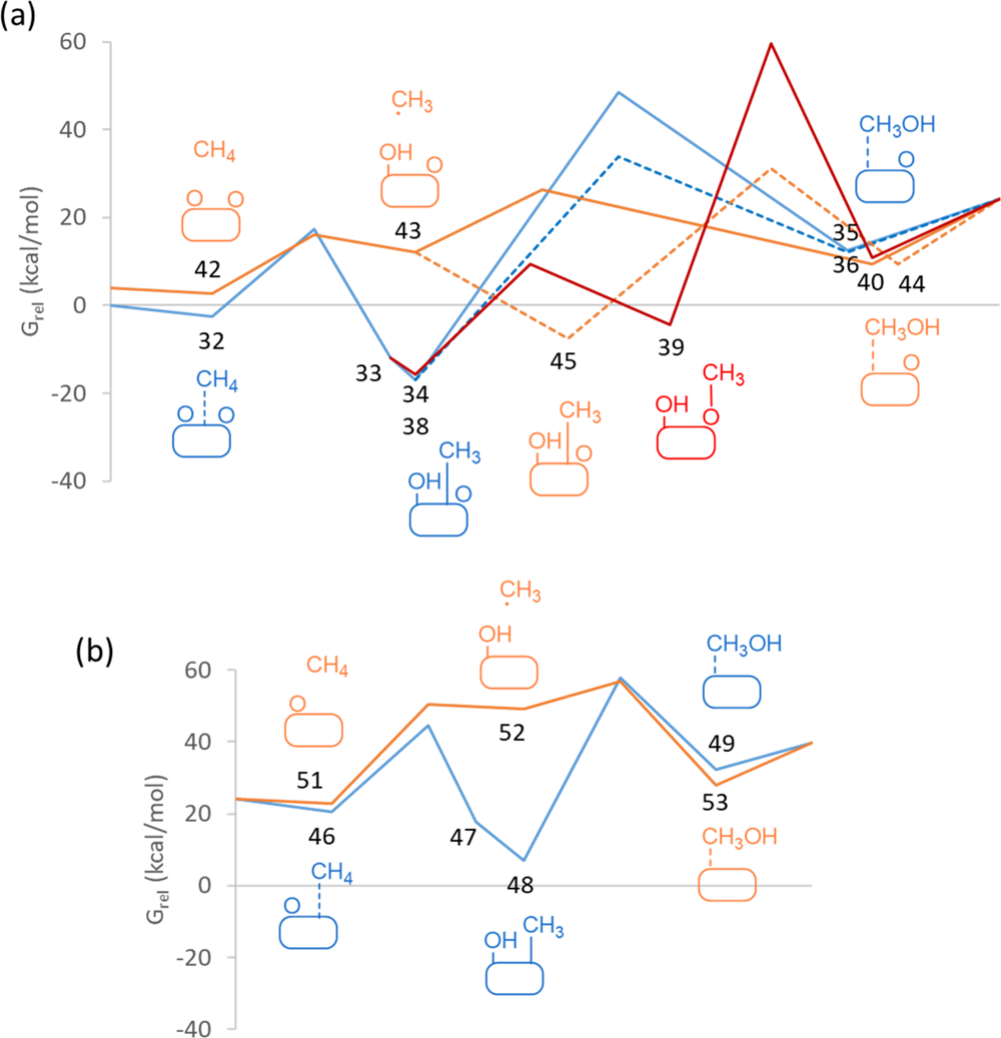
Energy profiles for CH_4_ oxidation to CH_3_OH on (a) Cu_7_-2O and (b) Cu_7_-O calculated at at 478 K. Langmuir–Hinshelwood and Eley–Rideal pathways are plotted as blue and orange lines, respectively. Formation of methoxy intermediates is depicted in red. The labels correspond to the optimized structures shown in Figures 7, 8, and 9. The origin of energies is in all cases the initial reactant structure **3**. The relative Gibbs energy values are summarized in Table S3.

The general reactivity trends obtained for Cu_7_ are summarized in Figure 10 and Table 1. As for Cu_5_, the LH pathways involving methyl adsorption on the metal cluster (blue lines in Figure 10) lead to high Gibbs activation energies of ∼50 kcal/mol, for the methanol formation step. Methoxy intermediates are energetically accessible but their subsequent transformation into methanol is kinetically forbidden (red line in Figure 10a). In contrast, direct reaction of methane from the gas phase following an ER pathway is less energetically demanding, and the calculated Gibbs activation energies are lower than 15 kcal/mol if the adsorbed O atom is bicoordinated (Figure 10a). However, if the adsorbed O is three-coordinated to the Cu cluster, an additional energy penalty must be paid to break one Cu–O bond, and the barriers increase to ∼30 kcal/mol (Figure 10b), making the process less viable.

**Figure 11:**
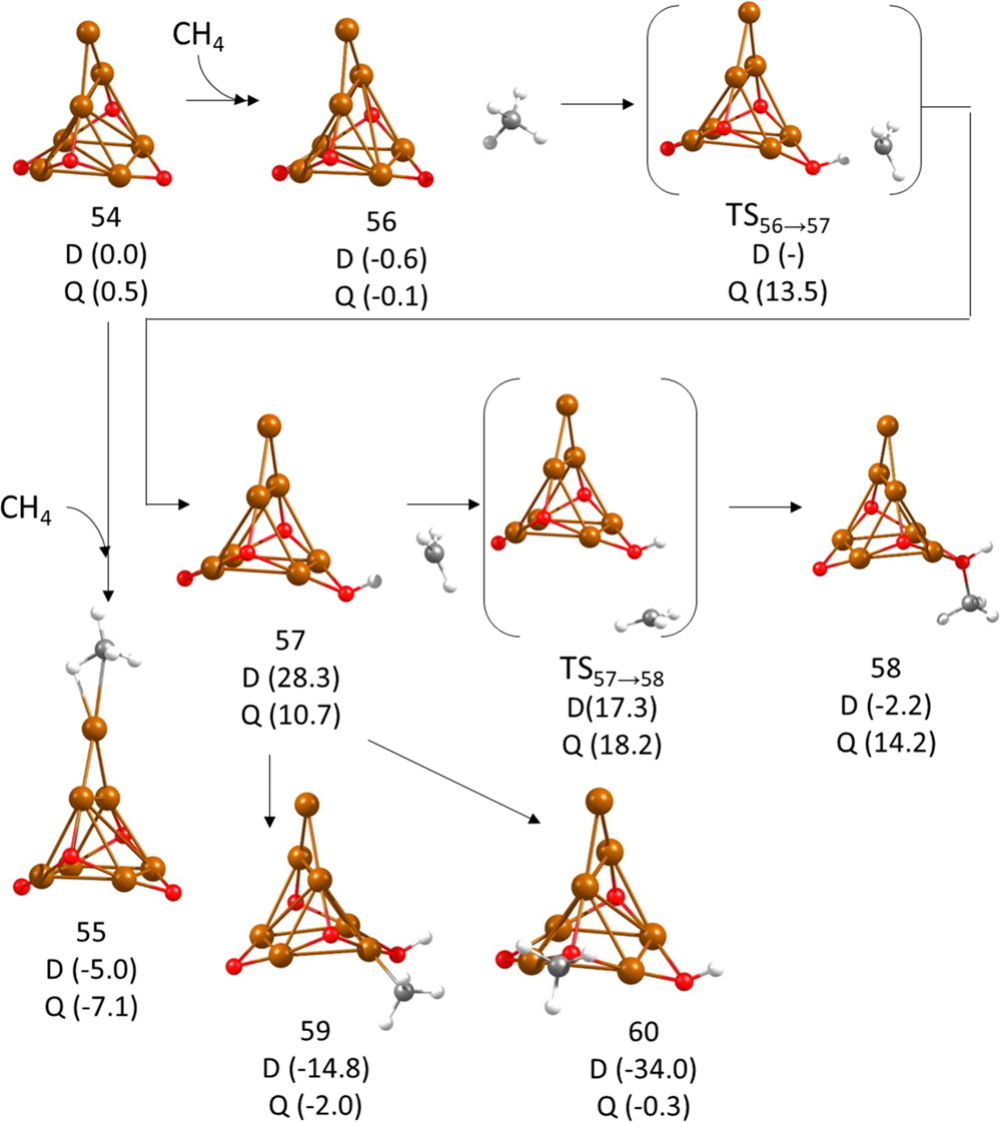
Optimized geometries of the structures involved in the mechanism of methane oxidation on partly oxidized Cu_7_-4O clusters. Relative energies of each structure in both doublet (D) and quadruplet (Q) states with respect to the initial reactant **54** in its most stable D state are given in kcal/mol in parenthesis below the structure label. Cu, O, C, and H atoms are depicted as orange, red, gray, and white balls.

Finally, taking into account the difficult reaction of methane with Cu_7_-O models and the strong affinity of O_2_ for copper, we studied the competitive adsorption and dissociation of an additional O_2_ molecule on the Cu_7_-2O system and its further reactivity with methane. Notice that, while all structures discussed up to the moment have only one unpaired electron and are therefore doublet (D), when a second O_2_ molecule is added to the system, the possibility to stabilize unpaired electrons increases and the difference in energy between the doublet (D) and quadruplet (Q) states decreases. The data in Figure S5 indicate that molecular O_2_ interacts strongly with structure **3** and dissociates with an activation energy of only 7.0 kcal/mol, generating a Cu_7_-4O system that contains two three-coordinated O atoms on the facets of the cluster and two bicoordinated O atoms placed at opposite edges (structure **54** in Figure 11). Since for structure **54**, the D state is only 0.5 kcal/mol more stable than the Q, the oxidation of CH_4_ on the Cu_7_-4O system has been investigated separately on D and Q potential energy surfaces (see Figure 11).

In both cases, methane interacts weakly with the Cu atom not in direct contact with O (structure **55** in Figure 11) and can also form a slightly less stable structure **56** via physisorption close to an O atom bicoordinated at the edge of the cluster. Hydrogen transfer from physisorbed CH_4_ to the bicoordinated O atom through transition state **TS56 → 57** is only possible on the Q potential energy surface with an activation energy of 13.0 kcal/mol and generates a Q methyl radical intermediate **57** that can easily react with the just-formed hydroxyl group through transition state **TS56 → 57**, yielding methanol (structure **58**) with an activation energy of 7.5 kcal/mol**.** This ER pathway therefore becomes competitive in Cu_7_ clusters, thanks to the additional bicoordinated O atoms that occupy bridge positions at the cluster edges. Interestingly, the possibility to form more stable Cu-methyl (structure **59**) or Cu-methoxy (structure **60**) intermediates, from which formation of methanol is difficult, is not completely excluded on these clusters but would require a change in the spin state of the system.

## 4 Conclusions

The mechanism of selective methane oxidation to methanol catalyzed by small copper clusters has been investigated in detail by means of DFT calculations. The influence of cluster size and shape has been analyzed by comparing the results obtained using Cu_5_ clusters with different morphologies (2D and 3D) with those provided by 3D Cu_7_. The O_2_ dissociation step has been assumed to be much faster than methane activation and therefore has not been included except in the particular case of 2D Cu_5_ clusters. Different Langmuir–Hinshelwood and Eley–Rideal pathways have been explored, and it has been found that in all cases, homolytic dissociation of a C–H bond of methane is assisted by adsorbed O atoms and results in formation of a hydroxyl group and a methyl species that must recombine in a second step to produce methanol. When the reaction follows a LH pathway, with all reactants and intermediates adsorbed on the copper cluster, the high stability of the hydroxyl and methyl intermediates makes their recombination energetically inaccessible. Formation of a methoxy intermediate by reaction of adsorbed O with methyl is energetically affordable, but its subsequent recombination to produce methanol is again kinetically forbidden. In contrast, an alternative ER pathway according to which methane reacts from the gas phase, producing a non-adsorbed radical-like methyl intermediate, is energetically favored if bicoordinated O atoms are available. Such bicoordinated O atoms are stabilized at the edges of 2D clusters as opposite to 3D clusters that preferentially stabilize three-coordinated O atoms at their facets. Thus, cluster morphology indirectly determines the feasibility of the methane oxidation reaction through the stabilization of different types of adsorbed O atoms. In addition, it is mandatory to avoid the adsorption of the methyl group on the copper clusters at any stage of the reaction. This could be achieved by selecting a proper support for the copper clusters, able to stabilize the desired 2D morphology without altering their electronic and catalytic properties, while blocking the non-desired interaction of methyl species with undercoordinated Cu atoms or by means of bimetallic copper containing clusters that preferentially stabilize bicoordinated O atoms while weakening the interaction with methyl species.

## Supporting Information Available

Optimized geometries and calculated interaction energies for CH_4_, O_2_, and O atoms adsorbed on Cu_5_-2D, Cu_5_-3D, and Cu_7_ clusters; optimized geometries and relative stability of CH_4_ adsorption at different sites in Cu_5_-2O and Cu_7_-2O models and of O_2_ adsorption and dissociation on a Cu_7_-2O system; net atomic charges on selected atoms of key structures involved in the mechanism of methane oxidation to methanol; Cartesian coordinates of all structures involved in the reaction pathways investigated (PDF)
