# Supplementary material for: Sub-nanometer Copper Clusters as Alternative Catalysts for the Selective Oxidation of Methane to Methanol with Molecular O2
Source: J Phys Chem A. 2022 Jul 21;126(30):4941–51. doi: 10.1021/acs.jpca.2c02895 (PMC10388348; doi:10.1021/acs.jpca.2c02895)
Supplement: Supplementary file 1 — jp2c02895_si_001.pdf [file jp2c02895_si_001.pdf]

Supporting Information

Sub Nanometer Copper Clusters as Alternative Catalysts for the Selective Oxidation of Methane to Methanol with Molecular O_2

*Mario Gallego, Avelino Corma and Mercedes Boronat**

Instituto de Tecnología Química (UPV-CSIC), Universitat Politècnica de València – Consejo Superior de Investigaciones Científicas, Avda. de los Naranjos s/n, 46022 Valencia, Spain.

*E-mail: boronat@itq.upv.es

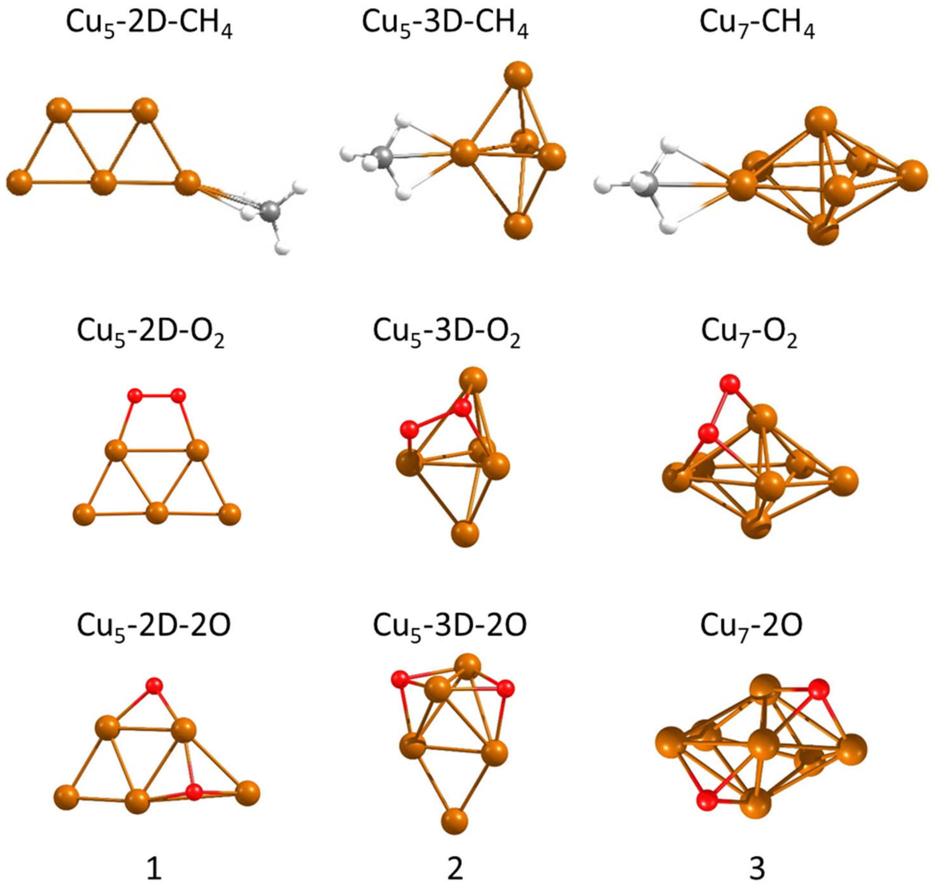

Figure S1. Optimized geometries of CH_4, O_2 and two O atoms adsorbed on Cu_5-2D, Cu_5-3D and Cu_7 clusters.

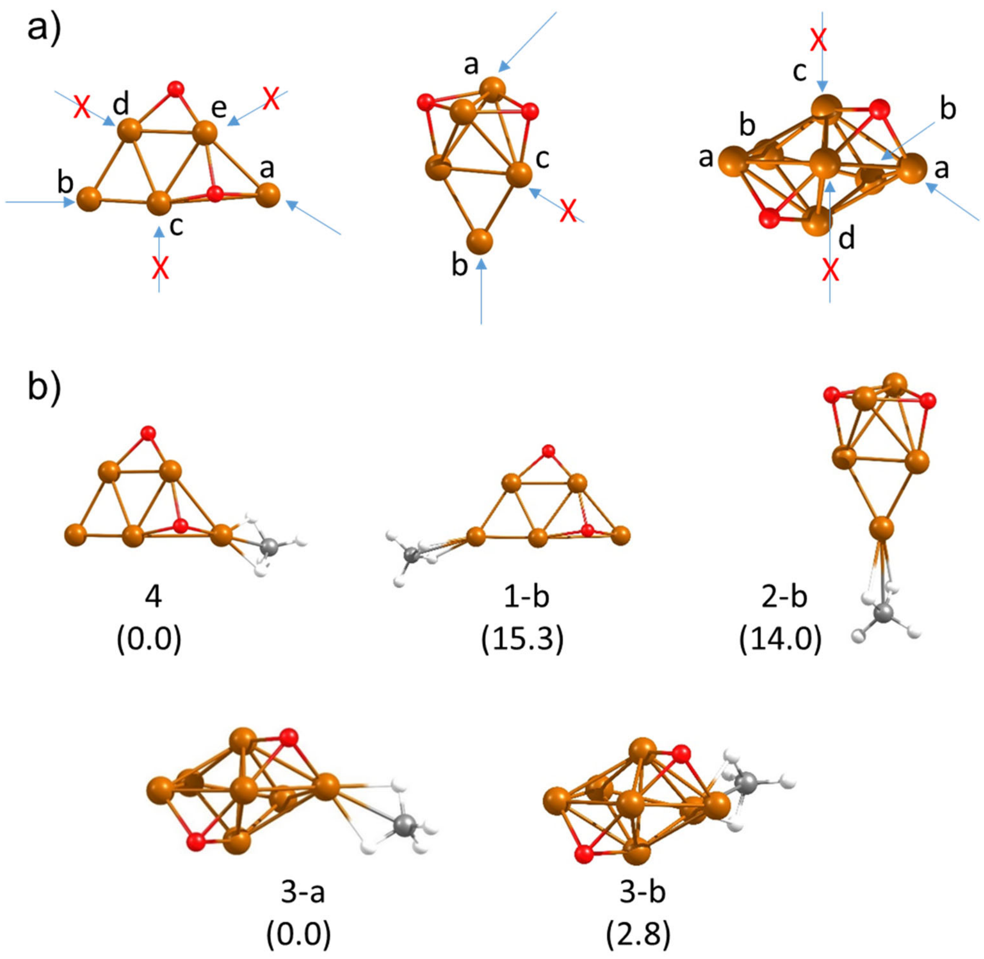

Figure S2. Possible sites for CH_4 adsorption on Cu_5-2O and Cu_7-2O systems (a), and optimized geometries of the most stable strucutres obtained, with the relative stability (in kcal/mol) given in parenthesis (b).

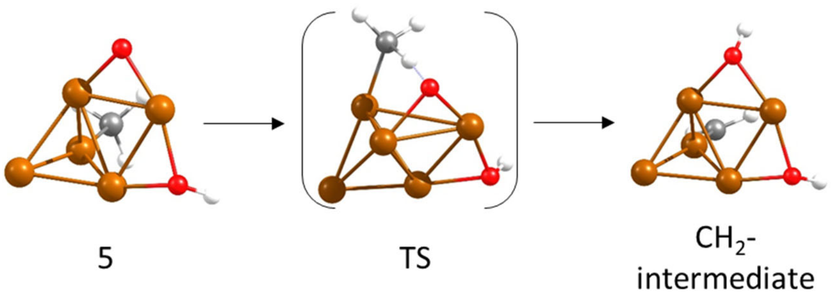

Figure S3. Optimized geometries of the structures involved in the secondary C-H bond dissociation in intermediate 5. The calcualted activation and reactio energies for this step are 25.1 and 5.6 kcal/mol, respectively. Cu, O, C and H atoms are depicted as orange, red, gray and white balls.

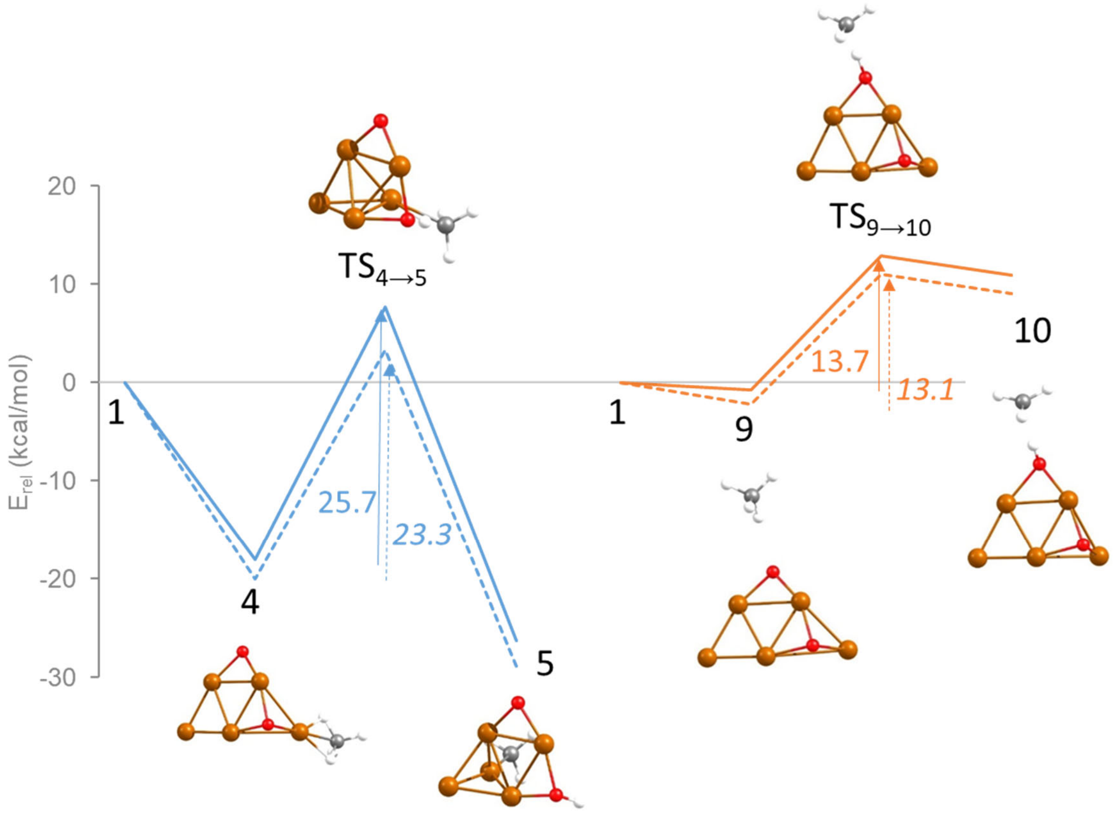

Figure S4. Energy profiles for the first steps of CH_4 oxidation on Cu_5-2O clusters following Langmuir-Hinshelwood (left, blue lines) and Eley-Rideal (right, orange lines) pahways. DFT and DFT-D3 energies are plotted as full and dashed lines, respectively. The optimized structures of the minima and transition states involved are shown together with the labels used for each structure in the main article.

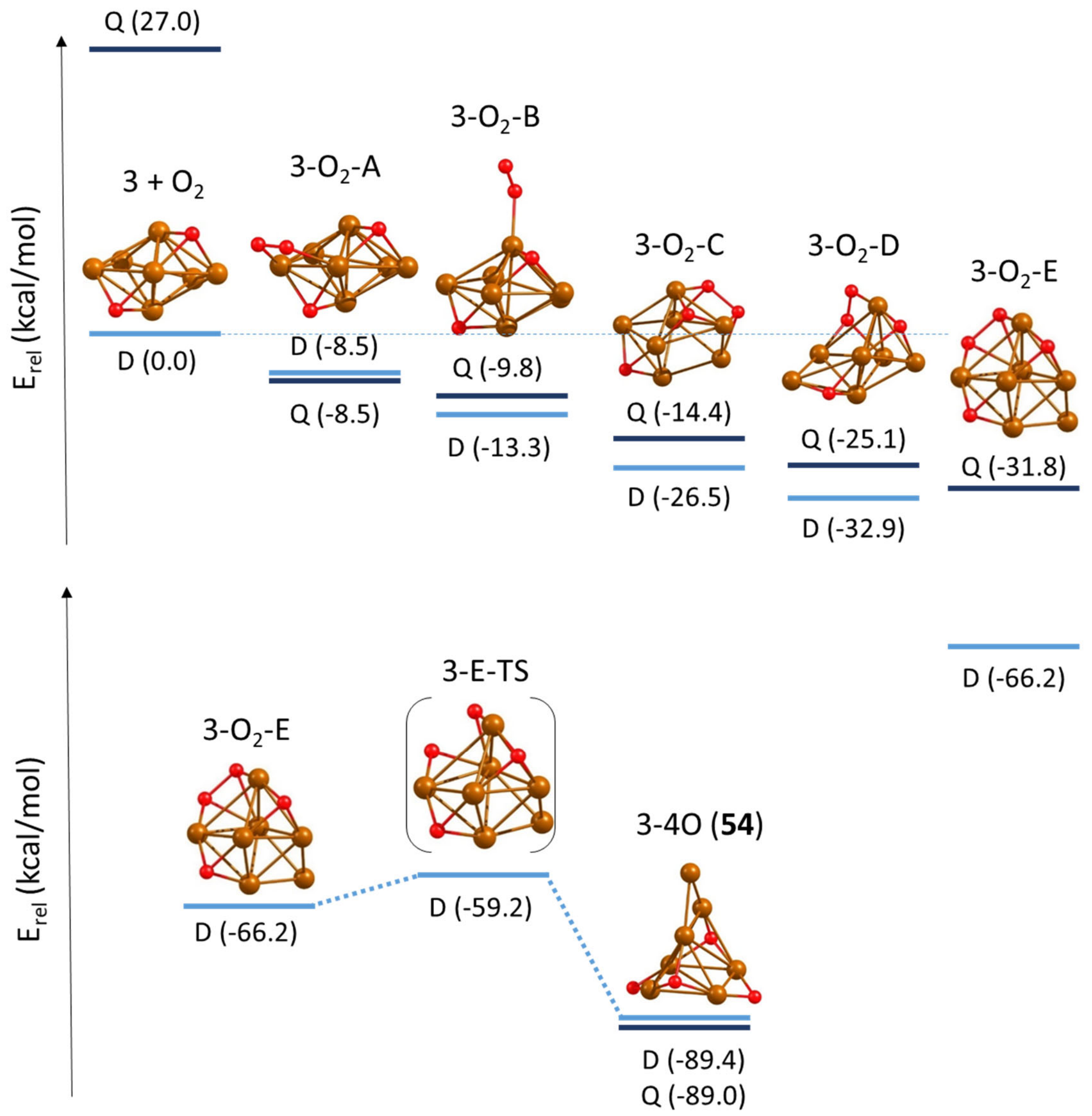

Figure S5. Optimized geometries of the structures involved in O_2 adsorption and dissociation on a Cu_7-2O system and relative energies of each structure in both doblet (D) and quadruplet (Q) states with respect to the initial reactant 3 + O_2 in its most stable D state. Cu, O, C and H atoms are depicted as orange, red, gray and white balls.

Table S1. Interaction energies (in kcal/mol) for CH_4, O_2 and two O atoms adsorbed on Cu_5-2D, Cu_5-3D and Cu_7 clusters. The corresponding structures are depicted in Figure S1.

	Cu_5-2D	Cu_5-3D	Cu_7
E_{int} CH_4	-2.6	-9.9	-1.9
E_{int} O_2	-46.3	-37.4	-27.4
E_{int} 2O[a]	-85.6	-86.2	-84.9

[a]Calculated with respect to separated Cu_n + O_2.

Table S2. Net atomic charges on selected atoms and spin state of key intermediates and transition states involved in the mechanism of methane oxidation to methanol.

	qO	qH	qC	qCH_3	$qH(CH_3)$	spin
4	-1.06		-0.18			D
TS4→5	-1.00	0.39	-0.46	-0.33	0.04	D
5	-1.59	1.00	-0.33	-0.28	0.01	D
TS5→6	-1.40	1.00	-1.03	-0.91	0.04	D
6	-1.66	1.00	0.49	0.69	0.07	D
TS5→8	-0.84	1.00	-0.01	0.09	0.03	D
8	-1.31	1.00	0.75	0.73	-0.01	D
9	-0.88		-1.10			D
TS9→10	-1.04	0.44	-0.14	-0.07	0.02	D
10	-1.60	1.00	-0.12	0.02	0.05	D
TS10→11	-1.49	1.00	0.12	0.20	0.03	D
11	-1.64	1.00	0.53	0.72	0.06	D
13	-1.60	1.00	-1.44	-1.39	0.02	D
TS13→14	-1.41	1.00	-0.05	0.15	0.07	D
14	-1.68	1.00	0.60	0.71	0.04	D
15	-1.95		-0.14			D
TS15→16	-1.01	0.36	-0.44	-0.33	0.04	D
16	-1.57	1.00	-0.25	-0.31	-0.02	D
TS16→17	-1.48	1.00	-0.15	-0.02	0.05	D
17	-1.64	1.00	0.58	0.70	0.04	D
19	-0.98		-0.04			D
TS19→20	-1.05	0.47	-0.15	-0.07	0.03	D
20	-1.58	1.00	-0.15	0.02	0.06	D
TS20→17	-1.51	1.00	0.02	0.10	0.03	D
21	-0.87		0.00			D
TS21→22	-1.04	043	-0.13	-0.05	0.03	D
22	-1.61	1.00	-0.14	0.02	0.05	D
TS22→23	-1.50	1.00	0.03	0.17	0.05	D
23	-0.69	1.00	0.60	0.72	0.04	D
24	-1.59	1.00	-0.41	-0.38	0.01	D
TS24→25	-1.39	1.00	-0.10	0.17	0.09	D

25	-1.67	1.00	0.54	0.71	0.06	D
29	-0.38		-0.05			D
TS29→30	-1.06	1.00	-0.14	-0.03	0.04	D
30	-1.06	1.00	-0.16	0.03	0.06	D
TS30→31	-1.10	1.00	-0.08	0.09	0.05	D
31	-1.77	1.00	0.84	0.79	-0.01	D
32	-1,02		-0.11			D
TS32→33	-1.02	0.37	-0.41	-0.35	0.02	D
33	-1.57	1.00	-0.32	-0.29	0.01	D
34	-1.58	1.00	-0.40	-0.36	0.01	D
TS34→35	-1.39	1,00	-0.03	0.16	0.06	D
35	-1.68	1.00	0.63	0.72	0.03	D
TS34→36	-1.50	1.00	-0.01	0.14	0.05	D
36	-1.67	1.00	0.52	0.70	0.06	D
38	-1.30	1.00	0.73	0.73	0.00	D
TS38→39	-1.32	1,00	0.69	0.74	0.02	D
39	-1.30	1.00	0.66	0.74	0.03	D
TS39→40	-1.48	1.00	0.13	0.35	0.07	D
40	-1.63	1.00	0.57	0.69	0.04	D
42	-0.84		-0.05			D
TS42→43	-1.03	0.45	-0.17	-0.07	0.03	D
43	-1.59	1.00	-0.16	0.02	0.06	D
TS43→44	-1.48	1.00	0.03	0.15	0.04	D
44	-1.67	1.00	0.55	0.69	0.05	D
45	-1,57	1.00	-0.28	-0.24	0.01	D
TS45→44	-1.43	1.00	0.01	0.15	0.05	D
46	-1.01		-0.16			D
TS46→47	-1.01	0.38	-0.36	-0.35	0.00	D
47	-1.57	1.00	-0.29	-0.30	0.00	D
48	-1.58	1.00	-0.37	-0.36	0.00	D
TS48→49	-1.43	1.00	0.04	0.12	0.03	D
49	-1.70	1.00	0.64	0.71	0.02	D
51	-0.99		-0.02			D
TS51→52	-1.59	1.00	-012	-0.07	0.02	D
52	-1.59	1.00	-0.12	0.02	0.05	D
TS52→53	-1.52	1.00	-0.06	0.11	0.06	D
53	-1.68	1.00	0.59	0.71	0.04	D
56	-0.87		0.02			D
TS56→57	-1.06	0.41	-0.13	-0.03	0.03	Q
57	-1.59	1.00	-0.20	0.04	0.08	Q
TS57→58	-1.52	1.00	-0.07	0.18	0.08	D
58	-1.61	1.00	0.46	0.69	0.08	D
59	-1.54	1.00	-0.18	-0.03	0.05	D
60	-1.31	1.00	0.62	0.72	0.03	D

Table S3. Realative energies (Erel) and Gibbs free energies (Grel) of all structures involved in the pathways depicted in Figures 1-10.

	Erel (kcal/mol)	Grel (kcal/mol)
1 + 2CH_4	0,0	0,0
2 + 2CH_4	-1,8	-1,6
4 + CH_4	-18,0	-11,2
TS4→5 + CH_4	7,7	13,0
5 + CH_4	-26,3	-17,5
TS5→6 + CH_4	32,3	43,2
6 + CH_4	7,9	17,6
7 + CH_4 + CH_3OH	21,2	26,1
TS5→8 + CH_4	15,4	25,6
8 + CH_4	-32,8	-21,7
9 + CH_4	-0,7	0,0
TS9→10 + CH_4	12,9	15,1
10 + CH_4	11,0	12,6
TS10→11 + CH_4	25,6	30,7
11 + CH_4	-5,4	5,1
12 + CH_4 + CH_3OH	13,9	19,7
13 + CH_4	-34,4	-28,0
TS13→14 + CH_4	41,7	46,8
14 + CH_4	10,0	16,3
15 + CH_3OH	14,5	21,6
TS15→16 + CH_3OH	32,6	42,3
16 + CH_3OH	-2,9	5,5
TS16→17 + CH_3OH	48,2	56,7
17 + CH_3OH	14,0	28,2
18 + 2CH_3OH	34,3	43,8
19 + CH_3OH	20,4	24,3
TS19→20 + CH_3OH	52,0	58,0
20 + CH_3OH	50,1	51,3
TS20→17 + CH_3OH	56,2	61,9
21 + CH_3OH	13,2	19,3
TS21→22 + CH_3OH	26,9	31,9
22 + CH_3OH	24,6	28,1
TS22→23 + CH_3OH	36,6	44,8
23 + CH_3OH	16,5	27,9
24 + CH_3OH	-16,4	-7,3
TS24→25 + CH_3OH	47,2	56,0
25 + CH_3OH	15,8	25,9
26 + 2CH_3OH	28,3	37,6
27 + CH_3OH	7,3	16,6
TS27→24 + CH_3OH	36,0	43,4
28 + CH_4	0,0	0,0
29	-0,5	-3,3
TS29→30	36,4	34,2
30	35,6	31,7

TS30→31	38,6	35,5
31	-33,1	-30,2
3 + 2CH_4	0,0	0,0
32 + CH_4	-5,1	-2,5
TS32→33 + CH_4	15,5	17,3
33 + CH_4	-12,6	-12,0
34 + CH_4	-23,3	-16,9
TS34→35 + CH_4	46,7	48,6
35 + CH_4	9,6	12,6
TS34→36 + CH_4	32,6	33,9
36 + CH_4	7,3	12,1
37 + CH_4 + CH_3OH	21,5	24,2
38 + CH_4	-19,1	-15,7
TS38→39 + CH_4	6,8	9,5
39 + CH_4	-5,8	-4,4
TS39→40 + CH_4	57,2	59,7
40 + CH_4	7,9	10,9
41 + 2CH_4	4,6	4,0
42 + CH_4	3,8	2,7
TS42→43 + CH_4	18,1	16,1
43 + CH_4	15,5	12,1
TS43→44	25,4	26,3
44 + CH_4	0,5	9,4
45 + CH_4	-10,0	-7,6
TS45→44 + CH_4	30,4	31,1
46 + CH_3OH	15,1	20,5
TS46→47 + CH_3OH	40,8	44,5
47 + CH_3OH	14,7	17,7
48 + CH_3OH	1,9	7,1
TS48→49 + CH_3OH	54,5	58,0
49 + CH_3OH	25,4	32,4
50 + 2CH_3OH	32,6	39,8
51 + CH_3OH	20,8	22,9
TS51→52 + CH_3OH	51,0	50,3
52 + CH_3OH	49,7	49,1
TS52→53 + CH_3OH	55,3	56,9
53 + CH_3OH	22,0	28,1

Table S4. Cartesian coordinates of all structures presented in this work, along with the number of the first figure in which the structure is shown.

Figure	Structure	Coordinates			
**1**	**1**	O	6.850260	10.532862	8.564171
		O	8.218837	7.452870	9.716548
		Cu	7.202450	8.988929	9.382654
		Cu	8.697195	8.114657	11.313302
		Cu	8.070071	10.047562	7.309118
		Cu	9.159925	8.092986	8.194111
		Cu	9.935356	9.111664	6.251046
**1**	**2**	Cu	7.538509	5.927300	8.925219
		Cu	6.397656	5.808185	10.970119
		Cu	8.739211	5.936024	10.969132
		Cu	9.807230	7.228555	8.960321
		Cu	9.932705	4.877048	8.899334
		O	10.497004	6.050762	10.334869
		O	8.947006	6.036874	7.697730
**1**	**4**	O	6.798751	10.525875	8.586898
		O	8.085407	7.411768	9.726289
		Cu	7.059291	8.925697	9.332709
		Cu	9.010521	8.188023	11.062006
		Cu	8.093914	10.078092	7.391227
		Cu	9.085325	8.055508	8.234039
		Cu	10.000886	9.156567	6.397782
		C	10.123994	9.089279	12.635101
		H	9.802868	9.137264	13.679337
		H	11.107017	9.543011	12.482328
		H	10.227653	7.993901	12.380834
		H	9.384535	9.691812	12.037950
**1**	**TS4→5**	O	8.450335	10.860559	8.447493
		O	6.578222	8.053498	9.291878
		Cu	7.109498	9.713916	8.729745
		Cu	9.077561	8.079815	10.302787
		Cu	9.559606	9.446232	8.292218
		Cu	8.069276	7.482688	8.242446
		Cu	10.459033	7.274378	8.470222
		C	7.686730	7.817326	11.776044
		H	7.023327	8.579139	12.205352
		H	8.658174	7.964794	12.312678
		H	7.345673	6.813498	12.055369
		H	7.175174	7.933263	10.480836
**1**	**5**	O	8.298622	10.919774	8.668201
		O	6.205997	8.161951	7.615547
		Cu	7.039203	9.784187	8.101556
		Cu	8.988076	7.571369	10.639240

		Cu	9.214402	9.421283	9.141874
		Cu	7.856541	7.499326	8.418067
		Cu	10.190392	7.288277	8.594563
		C	8.530925	7.836044	12.481080
		H	7.865892	8.700595	12.600364
		H	9.460073	7.990607	13.048071
		H	8.026402	6.918419	12.821425
		H	5.369362	7.954307	8.065932
**1**	**TS5→6**	O	8.408482	10.496169	7.629957
		O	6.014330	8.620766	9.749164
		Cu	7.245627	9.527467	8.582997
		Cu	8.704877	8.223239	10.123037
		Cu	9.730423	9.473097	8.298580
		Cu	8.757714	7.325100	7.951443
		Cu	10.848399	7.617553	9.258712
		C	6.959282	7.956110	11.177164
		H	6.334442	8.458592	11.922516
		H	8.027028	8.153516	11.656846
		H	6.744396	6.888317	11.112653
		H	5.422562	9.283291	10.166934
**1**	**6**	O	8.340319	10.353981	7.646291
		O	5.833115	8.869938	9.946367
		Cu	7.427491	9.383530	8.844231
		Cu	9.519106	8.560647	10.155114
		Cu	9.784045	9.312145	7.865978
		Cu	8.790045	7.187099	8.469613
		Cu	11.213516	7.614430	8.628141
		C	5.839625	7.798384	10.930784
		H	4.823571	7.664517	11.326056
		H	6.552579	8.013066	11.739097
		H	6.163112	6.897859	10.399533
		H	5.449000	9.667350	10.351868
**1**	**7**	O	8.315617	10.369925	7.644158
		Cu	7.534076	9.291113	8.870345
		Cu	9.502218	8.601401	10.147735
		Cu	9.787572	9.338978	7.843096
		Cu	8.759357	7.216525	8.444734
		Cu	11.175683	7.593888	8.659302
**1**	**TS 5→8**	O	6.407081	9.267949	8.932659
		O	7.231139	7.584195	12.106212
		Cu	6.696660	8.691559	10.665649
		Cu	8.937714	8.798204	9.235744
		Cu	7.067784	7.497246	8.500852
		Cu	8.337599	6.916697	10.624068
		Cu	9.121909	6.336561	8.492227
		C	7.838038	10.393869	8.331402
		H	7.582871	11.237514	8.970526
		H	7.374247	10.411664	7.346657

		H	8.959788	10.360858	8.174702
		H	7.730043	8.060699	12.793422
**1**	**8**	O	8.200685	10.788667	8.941833
		O	6.275323	8.024970	7.430533
		Cu	6.985736	9.591058	8.172759
		Cu	8.980686	7.005781	10.406530
		Cu	9.125010	9.119349	9.297528
		Cu	7.851648	7.294300	8.319483
		Cu	10.238133	7.249919	8.332242
		C	7.826534	11.726489	9.961178
		H	7.170103	12.498332	9.529040
		H	8.733171	12.217612	10.347452
		H	7.300371	11.249371	10.804499
		H	5.444170	7.756688	7.862148
**2**	**9**	O	8.234984	9.159238	8.147904
		O	7.780031	9.486519	11.665246
		Cu	9.903825	9.373962	8.836438
		Cu	11.700360	9.963565	10.213564
		Cu	9.588768	9.813140	11.179615
		Cu	6.594055	10.825008	11.538880
		Cu	7.637994	9.168775	9.828458
		C	7.971059	5.775249	6.794017
		H	8.708597	5.593560	6.003236
		H	6.968527	5.545469	6.414146
		H	8.191352	5.127653	7.651085
		H	8.015606	6.826389	7.105869
**2**	**TS9→10**	O	8.189195	8.412521	8.002632
		O	7.604313	9.201410	11.523738
		Cu	9.820883	9.035963	8.751184
		Cu	11.476355	10.044848	10.036256
		Cu	9.393436	9.722489	11.035012
		Cu	7.074548	10.868512	11.082376
		Cu	7.588589	8.674507	9.734802
		C	8.023630	6.050685	7.011641
		H	8.780408	6.079718	6.224853
		H	6.988074	6.029257	6.666869
		H	8.251090	5.401436	7.859500
		H	8.124819	7.375262	7.566298
**2**	**10**	O	8.127562	8.584258	7.941078
		O	7.596004	9.198200	11.537052
		Cu	9.788787	9.184057	8.748400
		Cu	11.483267	10.044502	10.083142
		Cu	9.399921	9.713131	11.079083
		Cu	7.140499	10.919049	11.230781
		Cu	7.563873	8.805634	9.722037
		C	8.025758	5.687075	6.875464
		H	8.807377	5.875431	6.143135
		H	6.984484	5.839111	6.601415

		H	8.269487	5.177779	7.804608
		H	8.136193	7.647211	7.631714
**2**	**TS10→11**	O	8.337414	8.859135	7.774681
		O	7.557796	9.285214	11.443774
		Cu	9.995066	9.285783	8.932802
		Cu	11.629177	9.862330	10.481915
		Cu	9.413932	9.662156	11.215121
		Cu	7.115800	11.043860	11.250476
		Cu	7.636242	9.032163	9.608792
		C	7.801910	9.698603	6.176547
		H	7.750686	10.720826	6.543023
		H	6.849786	9.215509	5.966154
		H	8.629159	9.465866	5.509012
		H	8.358638	7.912611	7.511975
**2**	**11**	O	8.584597	9.648465	7.576358
		O	6.555129	8.289172	10.386709
		Cu	8.948101	9.170459	12.738848
		Cu	10.047904	7.084864	12.403504
		Cu	8.127251	7.580715	11.190069
		Cu	7.009655	9.770374	11.508226
		Cu	7.536965	8.972546	9.050549
		C	8.621817	11.083393	7.312537
		H	7.582380	11.396330	7.180045
		H	9.186354	11.262241	6.388286
		H	9.069412	11.624509	8.156288
		H	9.497144	9.314710	7.652843
**2**	**12**	O	6.744020	7.722161	9.889577
		Cu	6.963637	9.389065	10.538284
		Cu	7.975544	11.269971	11.462686
		Cu	7.922416	7.121227	11.113372
		Cu	9.752525	7.037522	12.549968
		Cu	8.776354	9.140845	12.100058
**2**	**13**	O	7.894079	9.536632	7.714151
		O	6.581971	7.874185	10.776148
		Cu	9.341249	10.186447	8.743783
		Cu	9.340254	9.539942	10.979963
		Cu	8.324789	7.366532	11.341998
		Cu	7.103418	9.329250	11.889717
		Cu	7.223946	8.735492	9.290406
		C	10.817534	10.690528	9.918516
		H	11.705118	10.253132	9.434196
		H	11.017766	10.545604	11.016246
		H	10.844239	11.786768	9.805133
		H	7.302634	10.232872	7.378650
**2**	**TS13→14**	O	11.433181	11.444613	7.001790
		O	7.830555	9.605794	11.583803
		Cu	10.304625	10.925043	8.439236
		Cu	10.474645	8.929480	9.925341

		Cu	8.308003	7.988996	10.686233
		Cu	9.582978	9.248492	12.243800
		Cu	8.762069	10.466057	10.152492
		C	10.746463	12.948326	8.075562
		H	11.706950	13.149088	8.547142
		H	9.890950	13.014819	8.770618
		H	10.573278	13.509056	7.158332
		H	10.885003	11.523314	6.193989
**2**	**14**	O	10.861916	11.710413	6.916815
		O	7.798066	9.243133	11.797180
		Cu	10.039231	10.712265	8.571304
		Cu	10.475920	8.875096	10.085899
		Cu	8.478988	7.656672	10.960405
		Cu	9.611246	9.135501	12.412418
		Cu	8.552875	10.133025	10.298941
		C	11.194578	13.122491	6.933392
		H	11.733904	13.298870	7.869458
		H	10.288805	13.744952	6.912129
		H	11.845703	13.369371	6.083267
		H	10.411313	11.494933	6.080693
**4**	**15**	O	10.861916	11.710413	6.916815
		O	7.798066	9.243133	11.797180
		Cu	10.039231	10.712265	8.571304
		Cu	10.475920	8.875096	10.085899
		Cu	8.478988	7.656672	10.960405
		Cu	9.611246	9.135501	12.412418
		Cu	8.552875	10.133025	10.298941
		C	11.194578	13.122491	6.933392
		H	11.733904	13.298870	7.869458
		H	10.288805	13.744952	6.912129
		H	11.845703	13.369371	6.083267
		H	10.411313	11.494933	6.080693
**4**	**TS15→16**	O	9.272989	10.510669	8.674079
		Cu	10.481088	9.163885	8.099508
		Cu	11.364519	7.126810	9.045607
		Cu	9.072454	7.146369	8.278410
		Cu	9.711349	8.430446	10.234130
		Cu	7.978114	9.255419	8.212466
		C	9.346956	10.059302	11.428406
		H	9.348273	10.191241	10.055975
		H	9.210421	11.152291	11.376930
		H	8.456767	9.672821	11.947690
		H	10.246749	9.910611	12.045713
**4**	**16**	O	9.194825	10.600837	7.651016
		Cu	10.401446	9.160166	8.114145
		Cu	11.379492	7.302484	9.117669
		Cu	9.005937	7.243838	8.658254
		Cu	9.784049	8.610513	10.499416

		Cu	8.137972	9.482371	8.886728
		C	9.361090	9.340888	12.226674
		H	9.456229	11.445863	8.060766
		H	9.192885	10.425215	12.171329
		H	8.467189	8.852057	12.637348
		H	10.225905	9.137840	12.877867
**4**	**TS16→17**	O	9.157249	10.564178	9.235849
		Cu	8.819893	8.832143	9.633395
		Cu	8.255451	6.957974	8.074566
		Cu	7.260782	7.163627	10.244499
		Cu	9.528729	6.586531	10.066590
		Cu	8.790887	7.874348	11.946651
		C	11.578376	11.555883	9.025056
		H	9.064365	10.715936	8.277377
		H	11.199804	12.532067	8.738322
		H	11.668039	11.300437	10.075113
		H	11.912081	10.855031	8.265761
**4**	**17**	O	9.155675	10.560440	8.944750
		Cu	8.738450	8.717260	9.597080
		Cu	7.996138	6.887906	8.149296
		Cu	7.233299	7.117870	10.413182
		Cu	9.615156	6.548139	9.889096
		Cu	9.033965	7.804423	11.845892
		C	10.465898	11.138064	9.198428
		H	8.954598	10.622853	7.994742
		H	10.469540	12.191795	8.887103
		H	10.624174	11.066947	10.278812
		H	11.251006	10.576465	8.674100
**4**	**18**	Cu	6.420404	8.453924	8.204679
		Cu	6.421444	8.454722	10.610495
		Cu	8.673334	8.453158	11.457615
		Cu	8.018397	9.733322	9.504129
		Cu	8.019357	7.177636	9.503820
**4**	**19**	O	8.769131	9.716846	9.167990
		Cu	7.478765	8.474181	8.631482
		Cu	9.625946	8.163744	9.979867
		Cu	9.847643	8.889938	7.767932
		Cu	8.921553	6.636181	8.174615
		Cu	11.267940	7.065945	8.546919
		H	8.119571	13.071872	11.460563
		H	7.456807	13.468956	9.848841
		H	9.182420	13.771300	10.204618
		H	8.580647	12.086950	10.033829
		C	8.337111	13.096993	10.386232
**4**	**TS19→20**	O	10.727435	9.525030	9.521952
		Cu	9.482349	6.690747	9.015787
		Cu	9.677644	8.960176	7.968616
		Cu	8.985546	8.891340	10.151956

		Cu	7.543927	7.887478	8.461551
		Cu	7.559075	10.219731	8.558265
		H	10.940573	10.594031	9.626429
		H	10.224882	12.473698	9.748516
		H	11.743854	12.083159	10.734540
		H	11.851735	12.246799	8.894553
		C	11.232286	12.054036	9.771492
**4**	**20**	O	10.815085	9.447375	9.543202
		Cu	9.348244	6.576205	8.978524
		Cu	9.713203	8.871627	7.978026
		Cu	9.039159	8.773048	10.157563
		Cu	7.520667	7.941508	8.452754
		Cu	7.715484	10.270269	8.594281
		H	10.862465	10.429490	9.598185
		H	10.285048	12.868550	9.784347
		H	11.785830	12.312417	10.774323
		H	11.917651	12.531628	8.912150
		C	11.315225	12.522241	9.817049
**4**	**TS20→17**	O	10.080968	8.367211	9.768183
		Cu	11.060114	8.657968	6.578100
		Cu	9.614413	9.723064	8.331418
		Cu	9.368735	7.479479	7.959484
		Cu	8.784069	9.108157	6.170270
		Cu	7.299438	8.893549	7.983930
		H	9.328516	8.333157	10.392771
		H	11.043096	8.381040	12.185868
		H	12.175324	7.601955	10.922950
		H	12.015950	9.459992	11.013986
		C	11.645791	8.476674	11.286106
**5**	**21**	O	7.028214	7.036659	9.136558
		Cu	8.407433	6.730774	10.256861
		Cu	7.335764	5.999466	12.281823
		Cu	9.675542	5.854999	12.001804
		Cu	5.893995	6.756524	10.510613
		Cu	4.984348	5.914421	12.479106
		H	6.997849	9.552319	8.670349
		H	7.899918	11.061584	9.028364
		H	6.949084	10.924030	7.519259
		H	6.112235	11.053156	9.094413
		C	6.990092	10.645825	8.578810
**5**	**TS21→22**	O	7.042419	7.849006	9.155179
		Cu	8.399774	7.146636	10.248384
		Cu	7.314780	6.034304	12.075920
		Cu	9.652891	5.935699	11.778835
		Cu	5.914609	7.178846	10.500782
		Cu	4.961685	6.002322	12.256749
		H	7.029394	8.957539	8.959152
		H	7.934316	10.730777	9.134642

		H	6.957860	10.384108	7.604433
		H	6.089884	10.699581	9.204807
		C	6.999817	10.375775	8.695609
**5**	**22**	O	7.033330	7.713476	9.066953
		Cu	8.392563	7.025236	10.227162
		Cu	7.319433	6.028594	12.119309
		Cu	9.662101	5.952860	11.840156
		Cu	5.915735	7.058404	10.476583
		Cu	4.967183	6.023091	12.317669
		H	7.035457	8.697805	8.990881
		H	7.957056	10.979275	9.110823
		H	6.964088	10.573419	7.565895
		H	6.078711	10.952617	9.181167
		C	7.001425	10.774767	8.633951
**5**	**TS22→23**	O	7.074069	6.895333	8.806152
		Cu	8.406106	6.526959	10.301072
		Cu	7.286320	5.922301	12.339131
		Cu	9.619099	5.919098	12.194376
		Cu	5.933504	6.508750	10.445735
		Cu	4.950928	5.894934	12.466850
		H	7.056873	7.871919	8.689438
		H	6.000659	6.709969	6.662504
		H	7.849875	6.793979	6.532684
		H	7.031903	5.252388	7.145184
		C	6.970595	6.322679	6.966749
**5**	**23**	O	6.373041	8.156110	8.668303
		Cu	8.987511	6.438486	10.579529
		Cu	7.441314	5.868993	12.291729
		Cu	9.705347	5.380019	12.575502
		Cu	6.630769	7.041334	10.391125
		Cu	5.174198	6.319007	12.120202
		H	7.186851	7.935369	8.174205
		H	7.147108	10.027314	9.275366
		H	6.149941	10.038746	7.771312
		H	5.371797	9.802842	9.368156
		C	6.267298	9.601580	8.772338
**5**	**24**	O	6.303065	7.655776	8.978651
		Cu	7.733850	8.469784	9.890548
		Cu	6.905100	5.582157	12.625631
		Cu	8.205294	7.401450	11.905357
		Cu	6.258700	6.516557	10.551913
		Cu	5.073826	4.691023	11.431032
		H	5.502559	8.202541	8.890114
		H	9.637269	8.689378	11.969861
		H	10.119098	8.925401	10.302196
		H	9.054694	10.113211	11.139110
		C	9.250663	9.041602	10.970280
**5**	**TS24→25**	O	9.345169	8.693322	10.660781

		Cu	9.614889	8.599389	8.794247
		Cu	9.706866	9.081845	4.663805
		Cu	10.154851	9.936107	6.859646
		Cu	9.393717	7.729487	6.593367
		Cu	8.944184	6.870415	4.453510
		H	8.715008	7.985175	10.905232
		H	11.237793	7.216980	9.186498
		H	11.596472	8.362571	10.571683
		H	10.629087	6.837454	10.855713
		C	10.912128	7.624340	10.158023
**5**	**25**	O	9.353421	8.211387	10.422614
		Cu	9.526189	8.711616	8.434233
		Cu	9.766870	9.307828	4.365062
		Cu	10.047319	10.114649	6.603115
		Cu	9.210124	7.934911	6.237011
		Cu	8.933532	7.124697	4.063350
		H	8.412605	8.060025	10.627278
		H	9.922184	6.175865	10.361137
		H	11.183463	7.368813	10.803443
		H	9.917713	6.941064	11.995797
		C	10.132472	7.092094	10.929547
**5**	**26**	Cu	7.184636	8.641883	6.505744
		Cu	7.190835	8.641096	8.859690
		Cu	9.278388	8.640969	10.011681
		Cu	11.271806	8.639273	8.758990
		Cu	9.233722	8.639590	7.622924
**5**	**27**	O	8.693771	10.012510	8.758397
		Cu	7.566427	8.467603	8.644034
		Cu	9.197836	9.559125	10.428674
		Cu	9.921993	8.799061	7.966850
		Cu	8.825491	6.729479	7.626769
		Cu	11.097485	7.048943	6.989891
		H	9.264940	8.411771	13.082196
		H	9.087460	9.955285	12.207249
		H	10.665256	9.559059	12.937050
		H	10.193730	8.525297	11.562410
		C	9.800518	9.113936	12.437073
**5**	**TS27→24**	O	8.479799	11.113054	8.821959
		Cu	7.604551	9.605359	8.081987
		Cu	9.308293	8.808007	9.563902
		Cu	9.901737	10.554079	7.723467
		Cu	9.488416	8.256215	7.238144
		Cu	11.430798	9.377182	6.409001
		H	8.330171	10.077811	11.822686
		H	8.844410	10.550157	9.976291
		H	9.922396	10.841319	11.481075
		H	9.782711	9.099994	11.582637
		C	9.247159	10.014606	11.223818

**6**	**28**	Cu 9.232377 8.639238 7.645727<br>Cu 11.330916 8.639202 8.684557<br>Cu 9.454639 8.638738 10.043013<br>Cu 7.135649 8.640946 8.830544<br>Cu 7.179086 8.645819 6.516235<br>O 8.086518 8.642016 11.328566<br>O 6.855541 8.642621 10.685257
**6**	**29**	Cu 9.182158 8.981599 6.740896<br>Cu 11.388052 8.573201 7.412241<br>Cu 9.684712 7.780890 8.765478<br>Cu 7.235266 8.274977 7.967373<br>Cu 7.015403 9.427970 5.970923<br>O 8.472253 7.064921 10.007542<br>O 7.175156 7.332138 9.587563<br>H 8.499296 7.275550 12.679049<br>H 8.053235 8.514020 13.892773<br>H 8.205524 6.799941 14.381436<br>H 9.666500 7.741820 13.958246<br>C 8.606632 7.582112 13.726235
**6**	**TS29→30**	Cu 10.239577 9.504671 8.709710<br>Cu 12.169114 9.032695 9.986754<br>Cu 10.096197 8.560592 10.901310<br>Cu 8.043982 9.081536 9.566273<br>Cu 8.298926 10.043707 7.477773<br>O 7.760527 7.066139 11.480956<br>O 8.234350 8.461820 11.373233<br>H 7.664593 6.932743 12.546532<br>H 7.171386 7.684180 14.341870<br>H 6.764840 5.886354 14.083798<br>H 8.530104 6.412514 14.336898<br>C 7.508360 6.683279 14.070228
**6**	**30**	Cu 9.119065 8.709416 6.976273<br>Cu 11.373937 8.846450 7.665828<br>Cu 9.752282 8.408639 9.260309<br>Cu 7.363390 8.288074 8.550864<br>Cu 6.849993 8.668477 6.327714<br>O 8.049073 6.847653 10.904088<br>O 8.149353 8.228373 10.301252<br>H 8.175996 7.091048 11.862130<br>H 7.910290 8.591886 13.485060<br>H 8.002751 6.889715 14.282777<br>H 9.570914 7.732682 13.680489<br>C 8.486174 7.709384 13.756242
**6**	**TS30→31**	Cu 8.242739 10.251997 11.018543<br>Cu 7.731702 8.995820 12.950125<br>Cu 7.319499 8.046559 10.878490<br>Cu 7.799104 9.388274 8.827638<br>Cu 8.626721 11.538382 9.076105

		O	7.927537	6.511874	8.502527
		O	6.970552	7.699628	9.049690
		H	7.221838	6.048325	8.008529
		H	10.162742	5.132020	8.521691
		H	9.551389	5.176363	6.748965
		H	8.831017	3.934696	7.965261
		C	9.469229	4.790518	7.760719
**6**	**31**	Cu	7.010501	10.508761	10.915164
		Cu	6.781661	9.409163	12.988321
		Cu	8.160477	8.446192	11.383042
		Cu	8.280530	9.559617	9.108134
		Cu	7.049909	11.500928	8.773527
		O	8.611479	5.449738	8.949888
		O	8.849593	7.974094	9.774183
		H	8.705570	6.402411	9.238145
		H	8.417355	4.362117	7.231674
		H	9.317292	5.890645	7.019162
		H	7.537159	5.915030	7.203888
		C	8.465499	5.415351	7.541040
**7**	**3**	O	9.849027	9.661651	9.853506
		O	9.436711	10.967252	6.520348
		Cu	9.986405	10.593841	8.253556
		Cu	7.761896	10.266348	7.320227
		Cu	9.050006	8.370419	8.574489
		Cu	6.514968	8.766689	8.664786
		Cu	7.690758	7.773084	6.727545
		Cu	9.611315	9.109687	6.193720
		Cu	8.030953	10.190379	9.864601
**7**	**32**	Cu	9.610949	9.021839	7.061540
		Cu	9.952926	9.616351	9.579065
		Cu	8.092565	8.657601	11.048510
		Cu	6.591331	7.615758	9.487465
		Cu	7.691479	7.407222	7.294803
		Cu	9.081194	7.381604	9.200092
		Cu	7.726220	9.511152	8.638225
		O	9.924593	8.204736	10.784523
		O	9.292214	10.558017	8.115248
		C	10.796247	8.250338	5.261557
		H	10.610405	7.368281	4.640593
		H	11.714658	8.768691	4.969383
		H	9.939690	8.946389	5.111995
		H	10.925469	7.872382	6.307720
**7**	**TS32→33**	Cu	9.024293	7.701442	7.239890
		Cu	10.593801	9.266820	8.970153
		Cu	9.411075	9.216585	11.221063
		Cu	7.236577	8.364317	10.767839
		Cu	7.206491	7.086467	8.663297
		Cu	9.322086	7.287142	9.693254

		Cu	8.188099	9.425850	8.878081
		O	10.848997	8.222841	10.483562
		O	9.539930	10.084425	7.681237
		C	9.638589	8.552194	5.440966
		H	9.501716	7.484490	5.160937
		H	8.951123	9.119946	4.801428
		H	9.563977	9.318039	6.604251
		H	10.677124	8.793242	5.180416
**7**	**33**	Cu	8.765776	7.957601	6.764772
		Cu	10.293889	9.877542	9.549169
		Cu	9.826265	8.195242	11.538988
		Cu	7.656061	8.268808	10.611461
		Cu	7.081050	7.465631	8.407334
		Cu	9.440630	7.616578	9.079439
		Cu	8.088201	9.609412	8.500539
		O	10.989668	8.256598	10.087562
		O	9.131623	11.215543	8.888887
		C	9.625546	7.535228	5.103972
		H	9.505480	6.463006	4.897071
		H	9.137710	8.130170	4.317465
		H	9.456535	11.651413	8.080876
		H	10.691300	7.793193	5.170458
**7**	**34**	Cu	9.733706	11.589445	9.533051
		Cu	12.019428	10.217076	9.041445
		Cu	10.928556	8.149170	9.660310
		Cu	8.656637	7.693417	8.936057
		Cu	7.658117	10.111342	8.677108
		Cu	9.839958	9.544268	7.986524
		Cu	9.052512	9.370159	10.535849
		O	8.139088	11.062193	10.259888
		O	11.487829	12.041185	9.014778
		H	11.512223	12.442650	8.128010
		H	13.714843	8.741486	9.957014
		H	13.446905	8.448814	8.199424
		H	12.691714	7.425524	9.396617
		C	12.963796	8.497210	9.187698
**7**	**TS34→35**	Cu	8.484148	8.011136	7.383957
		Cu	7.043961	8.329353	9.370479
		Cu	9.514214	7.970290	9.542144
		Cu	11.144174	9.701908	8.946051
		Cu	10.126186	9.778672	6.711181
		Cu	8.727375	10.054154	8.664647
		Cu	10.949494	7.664944	7.600548
		O	9.849452	7.993610	6.050295
		O	5.254988	8.014311	9.786588
		H	4.771492	8.865267	9.774308
		H	5.730821	8.965280	11.789144
		H	7.519691	8.789968	11.469536

		H	6.466272	7.291946	11.691141
		C	6.519056	8.334708	11.382018
**7**	**35**	Cu	8.649316	7.977060	7.143170
		Cu	7.398288	8.451481	9.123726
		Cu	9.811232	8.068527	9.282998
		Cu	11.385863	9.764293	8.516885
		Cu	10.297766	9.685641	6.309154
		Cu	8.981006	10.108672	8.282064
		Cu	11.133514	7.633309	7.313269
		O	9.988912	7.862780	5.786560
		O	5.796836	8.509446	10.445464
		H	5.258195	9.314210	10.341946
		H	5.009276	8.115494	12.351883
		H	6.557789	9.042476	12.343874
		H	6.546927	7.302244	11.921813
		C	5.984272	8.239092	11.861014
**7**	**TS34→36**	Cu	11.538478	10.506105	10.020142
		Cu	9.251380	9.918903	9.229292
		Cu	8.878855	7.570810	9.402073
		Cu	10.534715	6.546569	11.061926
		Cu	12.261478	8.215431	11.315041
		Cu	9.930528	8.807155	11.265609
		Cu	11.140984	8.223109	9.156588
		O	12.786848	9.188149	9.780805
		O	10.086298	11.745806	9.751335
		H	9.696771	12.006430	10.610438
		H	10.254827	14.297641	9.892521
		H	9.405942	13.699551	8.341336
		H	11.257679	13.551874	8.511135
		C	10.289100	13.718309	8.972698
**7**	**36**	Cu	11.355767	10.506277	9.906110
		Cu	9.006773	9.523944	9.126518
		Cu	8.918883	7.148592	9.529039
		Cu	10.676924	6.550051	11.095054
		Cu	12.228428	8.400225	11.218285
		Cu	9.832172	8.789234	11.215565
		Cu	11.042216	8.105797	9.111304
		O	12.604406	9.206173	9.527577
		O	10.473132	12.251662	10.304694
		H	9.580936	11.935530	10.565563
		H	9.848841	14.158140	9.703843
		H	9.780498	12.898214	8.413984
		H	11.362369	13.534066	8.972607
		C	10.344483	13.275301	9.278943
**7**	**37**	Cu	8.630895	7.960013	7.114750
		Cu	7.441356	8.457583	9.109035
		Cu	9.818377	8.064655	9.294239
		Cu	11.374811	9.762544	8.521478

		Cu	10.283649	9.690101	6.314825
		Cu	8.983065	10.123893	8.285163
		Cu	11.120914	7.625815	7.325499
		O	9.992835	7.867161	5.792837
**7**	**38**	Cu	9.713632	12.021461	9.604293
		Cu	11.312263	10.124332	9.103596
		Cu	11.813223	7.884042	8.764801
		Cu	7.961051	7.675525	9.068882
		Cu	8.278917	9.945975	9.421454
		Cu	9.742323	8.725065	7.835218
		Cu	9.968515	8.359797	10.229771
		O	7.882735	11.808224	9.749231
		O	11.552384	12.024912	9.397679
		H	11.814952	12.487207	8.581300
		H	7.572449	11.776745	11.846801
		H	6.116065	11.942741	10.821345
		H	7.230401	13.326744	11.020697
		C	7.173806	12.230920	10.925451
**7**	**TS38→39**	Cu	8.508135	11.144889	9.109162
		Cu	10.944031	11.215640	9.091969
		Cu	12.058259	9.524326	7.781429
		Cu	9.859703	7.184326	9.470372
		Cu	8.647409	8.991475	10.310884
		Cu	9.727642	9.325765	8.124174
		Cu	11.197449	9.025815	10.014767
		O	7.507618	10.516429	10.644805
		O	11.475556	11.838093	10.726660
		H	10.742054	11.884909	11.366375
		H	5.811023	9.931847	11.692291
		H	5.630289	9.864824	9.915292
		H	5.649557	11.443532	10.753431
		C	6.081071	10.430370	10.747660
**7**	**39**	O	10.067017	12.634611	9.698070
		O	7.151740	9.821542	9.909214
		Cu	8.744659	9.481492	10.983227
		Cu	10.312161	8.361355	9.510774
		Cu	10.406122	10.848141	9.642208
		Cu	10.687835	9.581326	7.511067
		Cu	12.389218	9.523871	9.366828
		Cu	11.097592	9.361365	11.512319
		Cu	8.489154	9.597303	8.507708
		C	6.496621	11.097168	10.034867
		H	5.764305	11.213076	9.219635
		H	5.957903	11.138211	10.995147
		H	7.209288	11.938931	9.990851
		H	10.240309	12.976919	10.593593
**7**	**TS39→40**	O	9.591361	12.454321	9.749763
		O	7.543991	10.313336	9.934825

		Cu	8.843590	9.625937	11.057200
		Cu	10.319098	8.364965	9.590712
		Cu	10.519132	10.822547	9.595746
		Cu	10.758130	9.465567	7.527813
		Cu	12.460496	9.454427	9.351040
		Cu	11.229367	9.406974	11.521035
		Cu	8.546263	9.634387	8.536689
		C	7.414269	12.541645	9.810220
		H	7.401888	12.584005	8.726462
		H	6.581605	12.035492	10.289318
		H	7.769712	13.438499	10.312758
		H	9.795879	12.859883	10.613426
**7**	**40**	O	9.611089	12.615338	10.049487
		O	7.405926	9.522927	10.177126
		Cu	8.896118	9.210572	11.166578
		Cu	10.459818	8.306171	9.532461
		Cu	10.278075	10.724664	9.670295
		Cu	10.560872	9.583113	7.520797
		Cu	12.405525	9.699568	9.222561
		Cu	11.320883	9.399191	11.465642
		Cu	8.419495	9.312479	8.683457
		C	8.211873	12.871261	9.705730
		H	8.177832	12.928313	8.613101
		H	7.573282	12.045830	10.054153
		H	7.908097	13.834124	10.137731
		H	9.708494	12.726620	11.012266
**8**	**41**	O	9.520022	9.353053	10.186025
		O	10.927170	11.133490	7.420371
		Cu	6.803891	10.876675	7.990065
		Cu	7.881556	9.527245	6.373657
		Cu	8.980173	7.490511	6.863135
		Cu	10.308433	9.454201	6.718263
		Cu	8.452935	9.127774	8.679692
		Cu	9.029634	11.441423	7.383167
		Cu	10.500592	10.317046	9.038500
**8**	**42**	O	8.276435	9.294109	9.471191
		O	9.646403	10.818310	6.538730
		Cu	8.834733	10.373098	8.154141
		Cu	8.716447	9.242121	5.946626
		Cu	8.976898	7.989649	8.344033
		Cu	10.879159	9.448771	7.085246
		Cu	11.424823	7.474623	8.285632
		Cu	10.091303	7.248130	6.344802
		Cu	7.730721	7.119952	6.353921
		H	9.307595	9.510417	11.706041
		H	10.668985	10.387285	12.478905
		H	9.255566	9.892949	13.456224
		H	10.397322	8.657940	12.849936

		C	9.906628	9.611655	12.620023
**8**	**TS42→43**	O	8.627546	9.329709	9.927215
		O	9.647013	10.845954	6.812853
		Cu	9.160679	10.454474	8.558114
		Cu	8.573135	9.314583	6.403216
		Cu	9.262479	8.042922	8.613589
		Cu	10.927640	9.445339	7.126519
		Cu	11.648659	7.420192	8.114040
		Cu	9.953027	7.288352	6.463622
		Cu	7.622026	7.208042	6.887736
		H	9.149049	9.416142	10.923935
		H	10.501861	10.356450	12.055263
		H	8.922319	9.805780	12.837382
		H	10.203892	8.561445	12.368549
		C	9.777830	9.553842	12.207346
**8**	**43**	O	8.484503	9.323191	9.892413
		O	9.651664	10.848583	6.786603
		Cu	9.050218	10.443316	8.488964
		Cu	8.600012	9.320269	6.304987
		Cu	9.164568	8.034981	8.539718
		Cu	10.907860	9.446346	7.177359
		Cu	11.572430	7.416000	8.194749
		Cu	9.980006	7.294796	6.438141
		Cu	7.626418	7.208541	6.721936
		H	9.052438	9.382540	10.696330
		H	10.670969	10.397339	12.285501
		H	9.032719	9.833520	13.016884
		H	10.366265	8.571138	12.613747
		C	10.000860	9.594689	12.583406
**8**	**TS43→44**	O	9.638019	9.201403	10.059164
		O	10.261889	10.606331	6.699334
		Cu	10.076959	10.173474	8.476008
		Cu	8.625249	9.617616	6.493543
		Cu	9.229750	7.889567	8.327075
		Cu	10.950085	8.808433	6.580102
		Cu	11.027840	6.552277	7.276017
		Cu	9.142172	7.265477	6.004994
		Cu	7.100315	7.904494	7.062332
		H	10.455572	8.794913	10.415810
		H	8.555372	10.979783	11.292274
		H	8.354304	9.328647	12.119951
		H	9.953268	10.267672	12.283734
		C	9.014542	10.111466	11.755971
**8**	**44**	O	8.463518	9.864692	10.318103
		O	9.876762	10.614879	6.901383
		Cu	9.219389	10.194410	8.547278
		Cu	8.665511	9.298106	6.204737
		Cu	9.124090	7.626327	8.035508

		Cu	10.965072	9.041911	7.061651
		Cu	11.381787	6.793450	7.618449
		Cu	9.922257	7.235461	5.758934
		Cu	7.558554	7.220282	6.211809
		H	8.230741	8.911181	10.204457
		H	9.579334	11.070080	11.553178
		H	8.719470	9.737131	12.390284
		H	10.203875	9.391122	11.422017
		C	9.301917	10.013040	11.501669
**8**	**45**	O	8.702591	9.964974	9.895629
		O	9.734286	10.860481	6.523208
		Cu	9.246093	10.670159	8.280384
		Cu	8.568635	9.375081	6.256583
		Cu	9.166164	8.239202	8.827040
		Cu	10.975525	9.474437	6.946560
		Cu	11.573017	7.612089	8.239123
		Cu	9.834977	7.349577	6.664458
		Cu	7.505223	7.437455	7.036979
		H	9.362155	10.092620	10.601080
		H	9.230325	5.672227	9.445236
		H	7.899653	6.616230	10.215221
		H	9.588403	6.790428	10.814326
		C	8.960647	6.616106	9.929742
**8**	**TS45→44**	O	7.367316	9.740057	8.943634
		O	10.662476	11.336868	8.299348
		Cu	8.997653	10.616547	8.604866
		Cu	10.989386	9.798952	7.218969
		Cu	9.706831	8.238718	8.743087
		Cu	11.216266	10.098942	9.663725
		Cu	11.116139	8.012728	10.762487
		Cu	12.123957	8.091662	8.598568
		Cu	10.865866	7.519578	6.653544
		H	7.239104	9.719763	9.914240
		H	7.927516	7.102940	8.935270
		H	7.131181	7.933046	7.488861
		H	6.275623	7.833802	9.117076
		C	7.217534	7.862311	8.569485
**9**	**46**	O	10.012642	8.128697	5.863721
		Cu	11.077184	8.349997	7.440397
		Cu	9.441621	9.878647	8.558796
		Cu	9.709279	9.947454	5.960238
		Cu	11.552979	9.183358	9.584508
		Cu	9.766101	7.584293	9.375777
		Cu	7.541030	8.474628	9.199515
		Cu	8.656977	7.941106	7.201877
		C	9.545213	12.063838	5.346351
		H	9.137671	12.360208	4.374980
		H	9.784609	12.926392	5.975575

		H	8.740640	11.500255	5.898296
		H	10.499590	11.512547	5.166385
**9**	**TS46→47**	O	9.972236	7.889060	5.814289
		Cu	11.035078	8.295245	7.328244
		Cu	9.459338	9.652051	8.709358
		Cu	9.520880	10.180672	6.370173
		Cu	11.652596	8.847656	9.526151
		Cu	9.812531	7.332862	9.285701
		Cu	7.561493	8.153030	9.225596
		Cu	8.634624	7.871068	7.156484
		C	9.652384	10.122880	4.291850
		H	8.668522	10.583781	4.111482
		H	9.799706	9.407080	3.466511
		H	10.446205	10.877354	4.182402
		H	9.824329	8.958488	5.093046
**9**	**47**	O	10.149628	6.863320	6.140703
		Cu	11.043413	8.212502	7.258017
		Cu	9.458254	9.760410	8.337627
		Cu	9.619802	9.922422	5.966452
		Cu	11.603812	9.149578	9.334494
		Cu	9.738932	7.634836	9.417882
		Cu	7.512845	8.436086	8.992221
		Cu	8.686941	7.790760	7.066689
		C	9.544505	11.084972	4.436968
		H	8.554858	11.561377	4.407326
		H	9.704277	10.482440	3.530291
		H	10.332734	11.844568	4.532767
		H	10.176101	7.134987	5.204832
**9**	**48**	O	8.791140	10.591739	8.803416
		Cu	9.304372	8.803498	8.483796
		Cu	10.409240	9.110997	12.020663
		Cu	12.396604	9.596924	10.864331
		Cu	12.474871	11.612620	9.567269
		Cu	12.032879	9.507027	8.492193
		Cu	10.890916	7.925462	9.973750
		Cu	10.350054	10.546806	10.017408
		H	8.985715	11.200779	8.069086
		H	9.919206	6.648005	7.447741
		H	10.380394	6.264091	9.086093
		H	8.668369	6.444717	8.728361
		C	9.652571	6.879993	8.490305
**9**	**TS48→49**	O	8.226299	9.605673	8.897439
		Cu	10.469515	10.110899	7.989036
		Cu	10.829933	7.348622	10.897850
		Cu	11.616272	9.431103	11.703117
		Cu	10.115526	11.254470	11.821828
		Cu	11.535175	11.098072	9.894037
		Cu	11.943464	8.799658	9.343135

		Cu	9.678449	9.410678	10.271896
		H	7.825553	10.485137	9.067217
		H	7.579450	10.168281	6.828127
		H	9.341412	10.269168	6.507811
		H	8.564050	8.623542	6.845228
		C	8.536071	9.692797	7.049224
**9**	**49**	O	9.402837	7.637027	9.619832
		Cu	12.186838	9.489781	7.953120
		Cu	9.636468	11.401692	10.420833
		Cu	9.029586	11.826188	8.154143
		Cu	8.313405	9.898325	6.947108
		Cu	10.556546	10.664290	6.673943
		Cu	11.356107	11.561704	8.777737
		Cu	9.881933	9.565414	8.811371
		H	8.674694	7.296179	9.067323
		H	9.035283	6.369477	11.250188
		H	9.883655	7.911949	11.588729
		H	8.122334	7.925743	11.278076
		C	9.075515	7.443247	11.017658
**9**	**50**	Cu	9.632722	8.289267	8.899356
		Cu	9.558690	8.724856	6.479039
		Cu	7.596231	8.992846	7.870572
		Cu	11.095934	7.113715	7.424393
		Cu	11.505738	9.432330	7.810414
		Cu	10.648609	10.847585	6.087453
		Cu	9.365460	10.583416	8.084103
**9**	**51**	O	8.960859	9.729624	9.962573
		Cu	10.318705	8.403998	9.736972
		Cu	9.697032	9.815897	11.707772
		Cu	12.022285	9.679741	11.056919
		Cu	12.658747	11.633202	9.639636
		Cu	12.277087	9.490250	8.662141
		Cu	12.479803	7.494859	9.955400
		Cu	10.428234	10.885758	9.561177
		H	8.232134	9.593913	7.280966
		H	9.193758	9.956822	5.812375
		H	8.212603	8.463148	5.890459
		H	7.408409	10.055718	5.757567
		C	8.261380	9.517591	6.187187
**9**	**TS51→52**	O	8.865117	9.667980	8.932920
		Cu	10.159653	8.456986	9.704111
		Cu	9.816011	9.825739	11.802970
		Cu	11.956834	9.667972	10.916964
		Cu	12.489391	11.650052	9.605069
		Cu	12.199761	9.507722	8.563387
		Cu	12.315718	7.498310	9.872797
		Cu	10.259256	10.860786	9.544189
		H	8.688852	9.601575	7.868435

		H	9.310164	9.956845	5.954291
		H	8.289940	8.434733	6.235876
		H	7.493439	10.107202	6.292962
		C	8.403602	9.511368	6.366045
**9**	**52**	O	8.784291	9.684596	8.963264
		Cu	10.104791	8.475182	9.776210
		Cu	9.898556	9.830694	11.910236
		Cu	11.974336	9.667334	10.898937
		Cu	12.449557	11.639836	9.556341
		Cu	12.098955	9.497172	8.529860
		Cu	12.252526	7.493352	9.843666
		Cu	10.219847	10.863275	9.611533
		H	8.758926	9.611044	7.979604
		H	9.305215	9.924891	5.580243
		H	8.291470	8.379657	5.927568
		H	7.489213	10.075743	6.045233
		C	8.376893	9.463252	5.907411
**9**	**TS52→53**	O	8.942135	9.692297	9.505369
		Cu	10.480478	8.463123	10.042146
		Cu	10.774661	9.782667	12.156034
		Cu	12.585612	9.622715	10.701718
		Cu	12.745521	11.599701	9.313290
		Cu	12.079396	9.497258	8.370515
		Cu	12.580945	7.480593	9.572995
		Cu	10.574744	10.856088	9.892628
		H	8.817485	9.632854	8.536671
		H	6.404715	10.034476	9.066053
		H	6.745034	8.760495	10.391353
		H	6.948333	10.580426	10.766666
		C	6.818198	9.784877	10.040055
**9**	**53**	O	8.406166	10.281461	8.719108
		Cu	10.990477	7.953103	10.735929
		Cu	10.160008	9.729549	12.101607
		Cu	12.298012	10.004981	11.060719
		Cu	12.241290	11.262746	9.022312
		Cu	12.244683	8.877614	8.878512
		Cu	13.385328	7.867355	10.725846
		Cu	10.242667	10.095787	9.710153
		H	7.801427	9.652588	9.152745
		H	9.003200	10.839096	6.842680
		H	8.693702	9.084449	7.001902
		H	7.312851	10.246424	6.928738
		C	8.340884	10.087008	7.282907
**11**	**54**	O	9.838126	11.264896	9.567273
		O	9.822069	10.102851	6.165794
		O	7.215840	8.859798	10.037144
		O	6.282030	10.057334	6.784536
		Cu	9.922827	10.849025	7.848063

		Cu	7.989232	10.190781	6.334373
		Cu	8.433291	8.002830	8.853693
		Cu	6.545891	9.539865	8.453897
		Cu	9.759983	6.322597	7.922126
		Cu	9.825939	8.394760	6.913576
		Cu	8.488603	10.198766	9.975017
**11**	**55**	O	9.766855	11.423299	9.602401
		O	9.757960	10.316080	6.184635
		O	7.167247	8.976725	10.000366
		O	6.215467	10.266998	6.786226
		Cu	9.857435	11.044213	7.873372
		Cu	7.925187	10.417938	6.342433
		Cu	8.398112	8.191138	8.772721
		Cu	6.486666	9.709013	8.442461
		Cu	9.652088	6.449705	7.800505
		Cu	9.760826	8.580406	6.880038
		Cu	8.423149	10.336272	9.976508
		H	10.309368	3.128063	8.094197
		H	10.204746	4.515171	9.211417
		H	11.228489	4.607129	7.717490
		H	9.397276	4.521290	7.563881
		C	10.296304	4.224230	8.156350
**11**	**56**	O	10.155710	11.153413	9.754589
		O	9.974013	10.217992	6.288200
		O	7.308459	8.999931	10.123828
		O	6.456023	10.497872	6.973816
		Cu	10.174359	10.837335	8.012457
		Cu	8.163208	10.482098	6.500034
		Cu	8.415344	8.102721	8.863229
		Cu	6.688298	9.845041	8.600699
		Cu	9.541222	6.357608	7.797307
		Cu	9.810098	8.472361	6.920491
		Cu	8.710349	10.204965	10.121679
		H	4.460933	7.212660	6.032569
		H	4.988942	8.887742	5.675712
		H	5.128732	7.614268	4.421691
		H	3.516313	8.223589	4.899518
		C	4.524675	7.986161	5.257836
**11**	**TS56→57**	O	7.766515	13.018770	6.171441
		O	8.457765	9.918931	4.444968
		O	7.289623	10.528190	8.748201
		O	5.565858	8.510534	6.213507
		Cu	8.109670	11.551544	5.206030
		Cu	7.018319	9.100341	5.231643
		Cu	8.901683	9.752538	8.182990
		Cu	6.373352	9.434810	7.597555
		Cu	10.941517	8.797099	7.628160
		Cu	9.522121	9.430638	5.909728

		Cu	7.479869	11.879037	7.520877
		H	5.496316	7.377947	6.351255
		H	6.127125	5.596457	5.767266
		H	4.378333	5.785122	6.331653
		H	5.757140	5.829325	7.560585
		C	5.436895	5.970173	6.526244
**11**	**57**	O	7.955740	13.124562	6.172725
		O	8.437772	9.988064	4.442969
		O	7.312331	10.664655	8.740277
		O	5.466336	8.689891	6.219001
		Cu	8.208383	11.639297	5.207441
		Cu	6.959412	9.247008	5.227726
		Cu	8.876517	9.791101	8.182569
		Cu	6.341901	9.614510	7.597601
		Cu	10.868722	8.746376	7.623159
		Cu	9.471343	9.436667	5.908193
		Cu	7.598708	12.003011	7.520195
		H	5.436861	7.709751	6.352767
		H	6.145326	5.501109	5.747112
		H	4.359859	5.616151	6.324595
		H	5.758778	5.647757	7.580291
		C	5.424115	5.661547	6.545483
**11**	**TS57→58**	O	9.681416	11.889675	5.523716
		O	7.120006	9.583947	4.494525
		O	9.539028	10.074395	8.623375
		O	5.893396	10.318720	7.929795
		Cu	8.438155	10.823659	4.845324
		Cu	6.385011	9.955937	6.134769
		Cu	9.449731	8.571916	7.480448
		Cu	7.721334	10.227667	8.419551
		Cu	9.437886	6.582804	6.296061
		Cu	8.222100	8.333023	5.390033
		Cu	9.770518	11.092122	7.104554
		H	5.320483	9.649476	8.346372
		H	3.784697	11.901926	7.853889
		H	5.402681	12.836430	7.976234
		H	4.664944	12.047562	9.505738
		C	4.663812	12.181579	8.427597
**11**	**58**	O	8.407633	13.210889	6.997535
		O	9.027847	10.188547	5.071308
		O	7.545581	10.788784	9.572306
		O	5.731228	8.997453	6.684670
		Cu	8.733397	11.802762	5.947138
		Cu	7.465215	9.586501	5.802900
		Cu	9.092379	9.876276	8.956548
		Cu	6.656564	9.910046	8.241463
		Cu	11.027581	8.701345	8.461068
		Cu	9.848402	9.574295	6.668743

		Cu 7.941598 12.118807 8.333496<br>H 5.688628 8.027683 6.800854<br>H 4.225364 9.047811 5.212053<br>H 4.560852 10.587635 6.085857<br>H 3.655530 9.272570 6.921531<br>C 4.432442 9.506707 6.185262
**11**	**59**	O 8.418816 13.233497 7.138535<br>O 8.773181 10.216567 5.141325<br>O 7.559848 10.747818 9.625512<br>O 5.786258 8.983525 6.905927<br>Cu 8.556500 11.835780 6.031399<br>Cu 7.200278 9.545001 5.785350<br>Cu 9.134284 9.868405 9.109995<br>Cu 6.686231 9.786362 8.335594<br>Cu 11.035104 8.780545 8.365798<br>Cu 9.575563 9.646443 6.769747<br>Cu 7.958759 12.101116 8.435957<br>H 5.732012 8.012226 6.961368<br>H 7.263583 9.076506 3.330606<br>H 5.961588 10.268392 3.802487<br>H 5.708016 8.486566 4.121069<br>C 6.430884 9.299096 4.000060
**11**	**60**	O 8.336874 13.151739 6.912206<br>O 8.933153 10.135435 4.983537<br>O 7.315500 10.765732 9.493615<br>O 5.814160 8.898882 6.611440<br>Cu 8.656520 11.695812 5.934369<br>Cu 7.417698 9.429687 5.758004<br>Cu 8.957902 9.932335 8.581882<br>Cu 6.450593 9.767345 8.128902<br>Cu 10.934560 8.719783 8.376319<br>Cu 9.872466 9.457637 6.452675<br>Cu 7.776310 12.157251 8.267946<br>H 5.760500 7.933243 6.726461<br>H 6.834406 9.864627 11.313627<br>H 6.002812 11.421014 10.983074<br>H 7.740070 11.408814 11.429486<br>C 6.949372 10.871128 10.889664
